# De Novo Design of High‐Affinity Miniprotein Binders Targeting *Francisella Tularensis* Virulence Factor

**DOI:** 10.1002/anie.202516058

**Published:** 2025-10-21

**Authors:** Gizem Gokce‐Alpkilic, Buwei Huang, Andi Liu, Lieselotte S.M. Kreuk, Yaxi Wang, Victor Adebomi, Yensi Flores Bueso, Asim K. Bera, Alex Kang, Stacey R. Gerben, Stephen Rettie, Dionne K. Vafeados, Nicole Roullier, Inna Goreshnik, Xinting Li, David Baker, Joshua J. Woodward, Joseph D. Mougous, Gaurav Bhardwaj

**Affiliations:** ^1^ Molecular Engineering and Sciences Institute University of Washington Seattle WA USA; ^2^ Department of Medicinal Chemistry University of Washington Seattle WA USA; ^3^ Institute for Protein Design University of Washington Seattle WA USA; ^4^ Department of Biochemistry University of Washington Seattle WA USA; ^5^ Department of Bioengineering University of Washington Seattle WA USA; ^6^ Present address: Xaira Therapeutics Seattle WA USA; ^7^ Department of Microbiology University of Washington Seattle WA USA; ^8^ Present address: Sound Biologics Bothell WA USA; ^9^ Cancer Research @UCC University College Cork Cork Ireland; ^10^ Molecular and Cellular Biology Program University of Washington Seattle WA USA; ^11^ Howard Hughes Medical Institute University of Washington Seattle WA USA; ^12^ Microbial Interactions and Microbiome Center University of Washington Seattle WA USA

**Keywords:** De novo binder design, Deep learning‐guided design, High‑affinity protein binders, Protein engineering, Protein structure validation

## Abstract

*Francisella tularensis* poses considerable public health risk due to its high infectivity and potential for bioterrorism. Francisella‐like lipoprotein (Flpp3), a key virulence factor unique to Francisella, plays critical roles in infection and immune evasion, making it a promising target for therapeutic development. However, the lack of well‐defined binding pockets and structural information on native interactions has hindered structure‐guided ligand discovery against Flpp3. Here, we used a combination of physics‐based and deep‐learning methods to design high‐affinity miniprotein binders targeting two distinct sites on Flpp3. We identified four binders for site I with binding affinities ranging between 24–110 nM. For the second site, an initial binder showed a dissociation constant (*K_D_*) of 81 nM, and subsequent site saturation mutagenesis yielded variants with sub‐nanomolar affinities. Circular dichroism confirmed the topology of designed miniproteins. The X‐ray crystal structure of Flpp3 in complex with a site I binder is nearly identical to the design model (Cα root‐mean‐square deviation (RMSD): 0.9 Å). These designed miniproteins provide research tools to explore the roles of Flpp3 in tularemia and should enable the development of new therapeutic candidates.

## Graphical abstract

Hybrid physics and deep‐learning enabled design methods identified miniprotein binders to Flpp3 (Francisella‐like lipoprotein), a key virulence factor from *Francisella tularensis*. Multiple candidates with nanomolar to picomolar binding affinities were obtained using yeast surface display screening and further experimental optimization. X‐ray crystal structure of Flpp3‐miniprotein complex (PDB ID: 9NLT) closely matches the design model, validating the design approach.

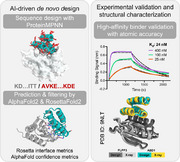

## Introduction

*Francisella tularensis (F. tularensis)*, the causative agent of tularemia (commonly known as “rabbit fever”), is a highly infectious Gram‐negative intracellular pathogen. As few as 10–25 bacteria can initiate a severe infection after subcutaneous or aerosol delivery, leading to serious clinical outcomes such as pneumonia, sepsis, and multiorgan failure.^[^ ^1^ ^]^ *F. tularensis* can leverage multiple immune evasion strategies to escape the phagosomes and replicate in the cytoplasm of macrophages and dendritic cells.^[^ ^2^ ^]^ Due to its high risk for weaponization and significant threats from natural outbreaks and bioterrorism, the Centers for Disease Control and Prevention (CDC) classifies *F. tularensis* as a Tier 1 Select Agent and a Class A bioterrorism agent.^[^ ^3^ ^]^ Despite considerable efforts, no approved vaccines are available for *F. tularensis*. While tularemia is currently treatable with antibiotics, such as fluoroquinolones, aminoglycosides, and tetracyclines, new medical countermeasures are needed to prepare for the natural and deliberate spread of emerging antibiotic‐resistant strains of *F. tularensis*.

The outer membrane lipoprotein, Francisella‐like lipoprotein (Flpp3), presents opportunities for developing novel research tools and therapeutic candidates against tularemia. It is a key virulence factor that plays critical roles in the spread and immune evasion of *F. tularensis*, and mutating the open reading frame encoding Flpp3 (FTT1416c) has been shown to significantly decrease its virulence.^[^ ^4^ ^]^ Flpp3 plays a key role in establishing the initial bacterial infection and promoting bacterial progression by evading the immune system.^[^ ^5^, ^6^, ^7^, ^8^ ^]^ Flpp3 interferes with host immune responses, notably by playing roles in extending the lifespan of neutrophils through a TLR2‐dependent mechanism that inhibits the apoptosis pathway, thereby promoting tissue destruction and bacterial dissemination.^[^ ^9^ ^]^ Previous studies have shown that *F. tularensis* can bind and activate plasma plasminogen, contributing to extracellular matrix degradation and potential systemic dissemination. Several outer membrane lipoproteins, including Flpp3, have been identified as potential plasminogen‐binding candidates, however, the specific role of Flpp3 in this process has not been established and remains to be experimentally confirmed.^[^ ^10^ ^]^ While some Flpp3 functions are well studied, the full scope of Flpp3's cellular localization, interactions with other proteins, and contributions to virulence remain unclear. Structurally, Flpp3 belongs to the bacterial lipoprotein family and shares low sequence and structural similarity to Bet v1 allergen proteins.^[^ ^11^ ^]^ While it contains a single‐helix membrane anchoring domain, studies suggest this may not be essential for membrane attachment as lipidation can anchor Flpp3 to the outer membrane.^[^ ^12^ ^]^ Flpp3 is also uniquely found in *F. tularensis*, making it an attractive target for therapeutic development, with the potential for developing ligands that disrupt its critical functions in immune evasion and bacterial dissemination.

The structure of Flpp3 has been characterized by X‐ray free‐electron laser (XFEL) and nuclear magnetic resonance (NMR) spectroscopy,^[^ ^8^ ^]^ providing opportunities for the structure‐guided design of Flpp3 inhibitors. However, there are still several challenges to targeting Flpp3 using rational approaches. First, no structurally‐characterized binding partners of Flpp3 have been identified to date. Typically, the key interactions from such binding partners provide the basis for rationally designing new therapeutic candidates. Second, no deep pockets on the surface of Flpp3 are available for targeting with small molecules. While the NMR structure shows an internal cavity that could act as a small molecule binding pocket, this cavity was not observed in the XFEL structure.^[^ ^8^ ^]^ Finally, the structural flexibility of Flpp3 also presents additional challenges for structure‐guided design efforts. Despite these challenges, we reasoned that recent advances in physics‐based and deep‐learning (DL)‐based design methods could enable the design of new Flpp3 binders. Such computational methods have recently been used to *de novo* design high‐affinity miniprotein binders with diverse shapes and sizes against various therapeutic targets of interest.^[^ ^13^, ^14^, ^15^, ^16^ ^]^ Miniproteins, with their compact size, high structural stability, and capacity for high potency and specificity, offer a compelling choice for targeting membrane‐bound proteins such as Flpp3.

Here, we leveraged *de novo* computational design methods to generate miniprotein binders targeting Flpp3. We identified multiple high‐affinity binders with low nanomolar to picomolar binding affinities against two different sites of Flpp3, providing avenues to explore the cellular localization of Flpp3, its functional roles in infection, and to develop new therapeutic interventions against tularemia.

## Results and Discussion

### Computational Design of Flpp3‐Binding Miniproteins

**Figure 1:**
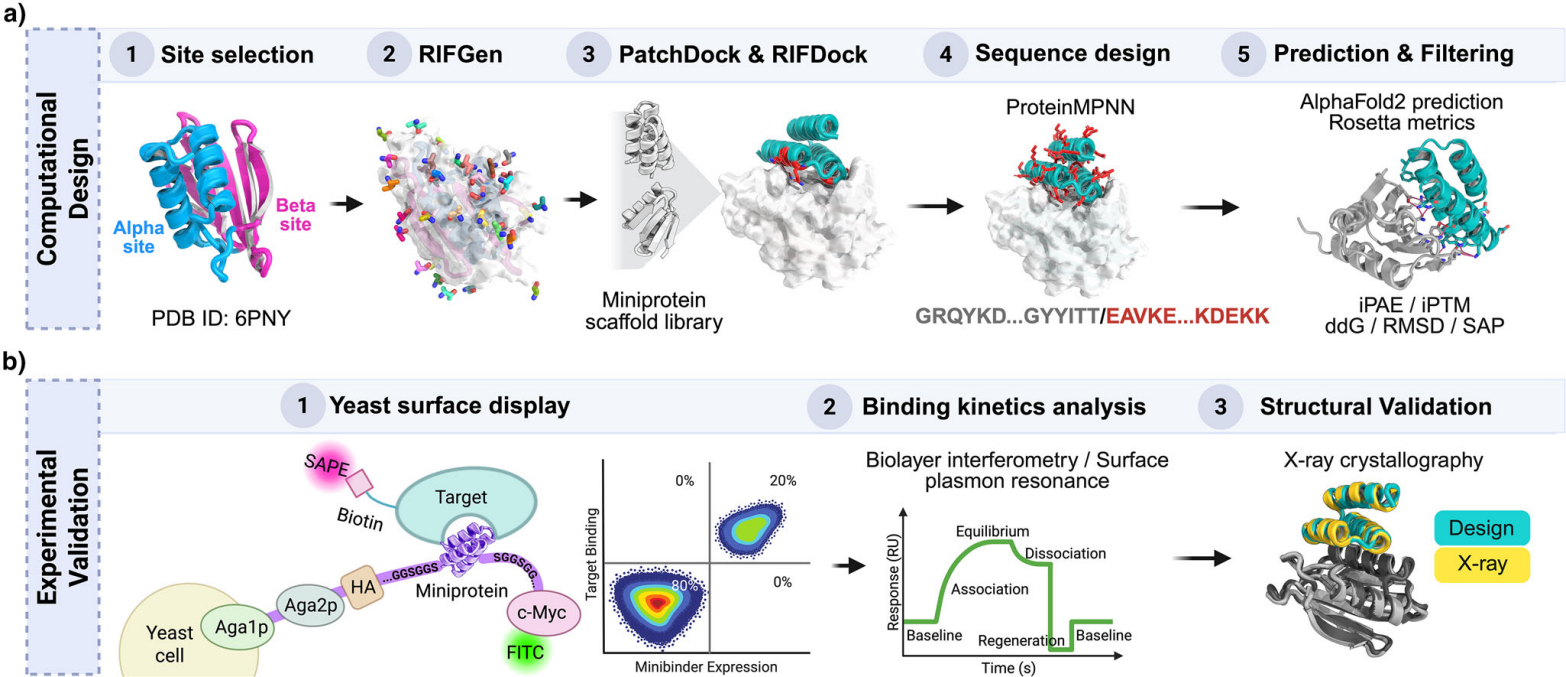
Design and screening pipeline for Flpp3‐binding miniproteins. a) Computational design workflow including site selection, RIFGen‐based docking,^[^ ^17^ ^]^ scaffold placement, ProteinMPNN‐based sequence design, and structure prediction and filtering with AlphaFold2 and Rosetta. b) Experimental validation pipeline including yeast surface display screening with FACS‐based enrichment and next‐generation sequencing of Flpp3‐binding miniproteins, followed by binding kinetics measurement via biolayer interferometry or surface plasmon resonance, and structural / biophysical characterization.

We used a combination of physics‐based and DL‐based methods to design, select, and optimize miniprotein binders against Flpp3. For initiating our design calculations, we used the structure of Flpp3 soluble domain (previously referred to as Flpp3sol,^[^ ^8^ ^]^ PDB ID: 6PNY), which is composed of six β‐sheets and two α‐helices. Since the structural basis of Flpp3's function as a virulence factor is largely unknown, particularly regarding the parts of the structure involved in interaction with the membrane or other interacting proteins to form potentially large molecular weight complexes, we decided to design minibinders against both faces of Flpp3 structure. We defined the electronegative face of Flpp3, dominated by the α‐helices, and hypothesized to interact with the inner leaflet of the outer membrane as the site I (or “α‐site”). The opposite face formed by the β‐sheets and exhibiting a relatively more electropositive character, was defined as the site II (or “β‐site”) (Figure 1a).

To design miniprotein binders against the selected sites, we used a three‐step computational pipeline (Figure 1). First, we used the Rosetta Rotamer Interaction Field Docking (RIFDock)^[^ ^13^ ^]^ approach to select miniprotein backbones with a high likelihood of making favorable interactions with Flpp3 from a pre‐enumerated library of miniprotein scaffolds (Figure 1a). The initial step in the Rosetta RIFDock approach, Rotamer Interaction Field Generation (RIFGen),^[^ ^13^ ^]^ involves docking disembodied amino acids against the selected target site and calculating target binding energies. These docked amino acids then act as a rapid lookup table for estimating the target interaction energy achievable by a miniprotein scaffold based on its backbone coordinates and provide a means to select putative binders even before the sequence has been redesigned. As no structurally characterized binders or hotspot motifs are known for Flpp3, a motif‐guided docking approach could not be applied. Instead, we adopted a scaffold‐based strategy. In this approach, PatchDock^[^ ^18^ ^]^ was first used to generate global placements of scaffold backbones relative to the target surface to identify positions where the scaffold and target achieve an optimal shape complementarity. These placements were then refined with RIFDock, which uses the rotamer interaction fields generated by RIFGen to optimize sidechain placements and assess the potential for favorable interactions. We used a scaffold library of 43724 pre‐enumerated miniproteins composed of 25–65 amino acids and predicted to fold into diverse shapes. Overall, through this hierarchical docking approach using PatchDock placements refined with RIFDock, we generated and selected 500000 conformations for further sequence design and in silico evaluation. Next, we used ProteinMPNN,^[^ ^19^ ^]^ a deep learning‐based tool for amino acid sequence design, to redesign the amino acid sequence of the selected minibinder backbones and improve the shape and chemical complementarity between the designed minibinders and Flpp3. We chose ProteinMPNN for sequence design over conventional approaches like Rosetta FastDesign (with RIFGen amino acids kept fixed) as prior work had shown that ProteinMPNN provides sequences with improved affinity, solubility, and overall success rates over traditional approaches.^[^ ^15^, ^19^, ^20^, ^21^ ^]^ Since ProteinMPNN does not allow backbone movement, we performed two iterative rounds of sequence design with ProteinMPNN followed by energy minimization using the Rosetta FastRelax protocol to allow for some backbone changes and achieve higher sequence diversity.^[^ ^22^ ^]^ Finally, all designed minibinders were filtered based on the Rosetta metrics for interface quality and by re‐predicting the structures of designed complexes using AlphaFold2 (AF2)^[^ ^23^ ^]^ to assess if designed models are confidently predicted to fold and bind as their designed conformations (Figure 1a). Newer structure prediction tools like AlphaFold3 (AF3)^[^ ^24^ ^]^ and RoseTTAFold All‐Atom (RoseTTAFold‐AA)^[^ ^25^ ^]^ should provide a better and improved performance in filtering; however, they were not available at the time and were not tried in this work. Specifically, we filtered designs based on AF2 pLDDT (> 90 for α‐site designs and > 80 for β‐site designs), interface PAE less than 6, Rosetta ddG values (< ‐40 kcal mol^−1^ for α‐site designs and < ‐35 kcal mol^−1^ for β‐site designs), and spatial aggregation propensity (SAP) scores (< 30 for α‐site designs and < 35 for β‐site designs). After the in silico filtering steps, we selected 15000 α‐site design models and 8817 β‐site design models for experimental screening and characterization.

### Yeast Display Screening and Biochemical Characterization

**Figure 2:**
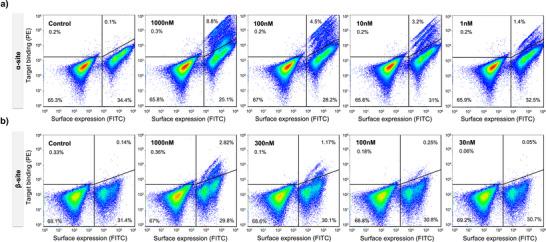
Yeast surface display screening of α‐site and β‐site libraries against Flpp3. a) Binding profiles of the α‐site library at decreasing concentrations of Flpp3 (1000, 100, 10, and 1 nM). A distinct population of Flpp3‐binding cells is observed even at the lowest concentration (1 nM), indicating high‐affinity binders. b) Binding profiles of the β‐site library at decreasing concentrations of Flpp3 (1000, 300, 100, and 30 nM). The binding signal at 30 nM is comparable to the negative control (no Flpp3), suggesting weaker binding relative to the α‐site.

We screened the selected α‐site and β‐site miniproteins for binding to Flpp3 using yeast surface display (YSD) and fluorescence‐activated cell sorting (FACS). Synthetic oligonucleotides encoding for the designed minibinders were commercially purchased and cloned into a pETCON3 expression vector for display on the surface of the yeast cells (see Methods for details) (Figure 1b). The α‐site and β‐site design libraries were ordered, cloned, and screened separately. After an initial expression sort to collect the yeast cells expressing the designed minibinders, a round of binding sort was conducted with 1 µM of Flpp3 to collect yeast cells that bind fluorescently labeled streptavidin‐tetramerized Flpp3. We observed a clear binding signal at 1 µM for both α‐site and β‐site libraries (Figure S4). To identify and select for the higher‐affinity binders among this initial binding population of cells, the binding cells from the initial binding sorts were grown and sorted again with decreasing concentrations of monomeric Flpp3: 1000, 100, 10, and 1 nM for the α‐site library and 1000, 300, 100, and 30 nM for the β‐site library (Figure 2). The α‐site library showed a clear population of Flpp3‐binding cells even at 1 nM (Figure 2a), but the signal for the β‐site library screened with 30 nM Flpp3 returned to levels comparable to the negative control (i.e., sorting with no Flpp3) (Figure 2b). After sequencing the binding cells from different sorting experiments using next‐generation sequencing (NGS), we observed several designs with promising enrichment ratios and estimated on‐yeast binding affinities as calculated from the counts of each sequence across the experiments (see Methods and Supporting Data Files). Since it was not feasible to test hundreds of promising candidates by individually expressing them in *Escherichia coli*, we focused on expressing and validating four promising binder candidates for the α‐site (ASD1–4) and one additional binder (BSD1) for the β‐site based on their enrichment ratios, raw counts in the NGS data collected at low‐target concentrations, and estimated on‐yeast binding affinities.

**Figure 3:**
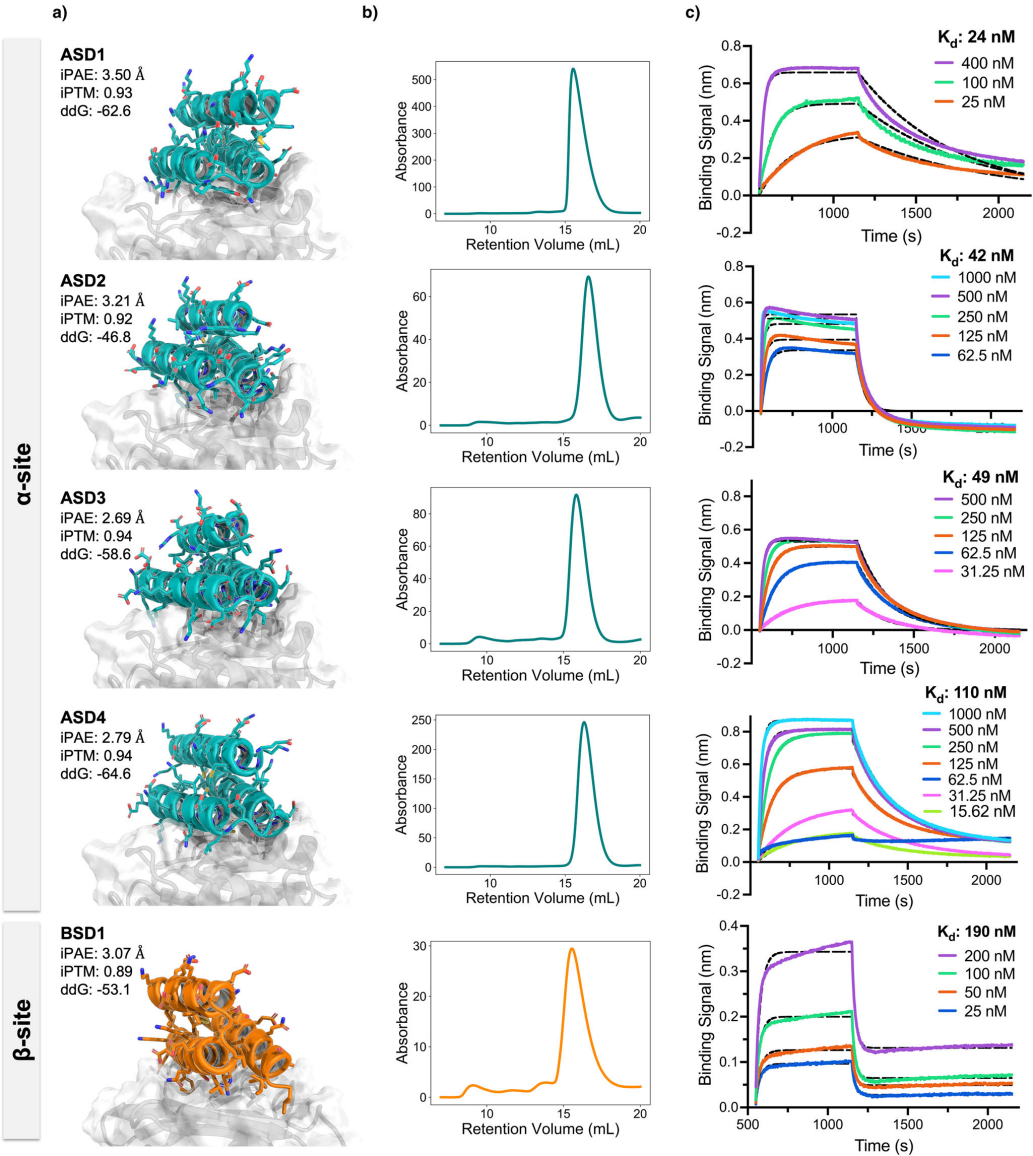
Design models and biochemical characterization of Flpp3‐binding miniproteins. a) Design models of the five binders (four for α‐site in teal and one for β‐site in orange). Flpp3 shown in gray. b) Size‐exclusion chromatography (SEC) profiles of the purified binders expressed in *E. coli*, demonstrating monodisperse peaks consistent with the monomer size of the minibinders. c) Bio‐layer interferometry (BLI) binding curves for the designed binders against biotinylated Flpp3. Calculated K_D_ for each binder reported on the plots.

The design models for all five binders from YSD screening have a three‐helix topology with two helices making extensive contacts with the Flpp3 surface (Figure 3a). Each putative binder demonstrated excellent computational metrics, such as AF2 Interface Predicted Aligned Error (iPAE) < 3.5, and Rosetta ddG < ‐40 kcal mol^−1^ (Figure 3a), indicating favorable binding interactions. We also compared the structure prediction confidence metrics from both AF2 and AF3 for these five selected binders against metrics for all designs in the library screened using yeast surface display. All five binders show excellent metrics and feature among some of the best scoring designs, demonstrating high pLDDT and low min interface PAE (Figure S5). Notably, there are several other designs that also show similarly good or even better scores as the five selected designs, suggesting that filtering by these metrics enriches for binders and improves the likelihood of experimental success but does not correlate perfectly with the real binding affinities, as has been observed in other studies as well.^[^ ^15^, ^26^ ^]^

To confirm the binding in solution (in addition to the target binding on yeast surface) and determine binding affinities, we expressed the five putative binders in *E. coli* and purified them using immobilized metal affinity chromatography (IMAC) and size‐exclusion chromatography (SEC). All binders expressed well with a single peak corresponding to the monomer size on SEC (Figure 3b). Next, we used biolayer interferometry (BLI) to confirm the binding of the miniproteins to the biotinylated Flpp3. Biotinylated‐Flpp3 was immobilized at 50–100 nM concentrations on streptavidin‐coated biosensors, and binding kinetics were measured by monitoring association and dissociation across a series of miniprotein concentrations in BLI assay buffer. All four α‐site designs showed binding to Flpp3 with binding affinities (*K_D_*) ranging between 24–110 nM (Figure 3c), with designs ASD1, ASD2, and ASD3 showing *K_D_* < 50 nM and the design ASD1 showing the best binding affinity (*K_D_*: 24 nM) for the α‐site. The β‐site design, BSD1, also showed promising binding to Flpp3 with a *K_D_* of 190 nM. The better success rate, in silico metrics, and affinities of α‐site designs compared to β‐site designs is likely a result of the differences between the two target surfaces. While the α‐site is a well‐defined concave pocket formed by helices, the β‐site is a relatively flat surface, which typically makes for a more challenging target for binder design.

Computational models for all four α‐site binders (ASD1‐4) have similar structural features and show similarity in binding mode and interactions. ASD1 and ASD4 show very similar three‐helix fold, binding mode, and interface contacts. ASD2 also has the same three‐helix fold and targets the same region of Flpp3 but shows a slightly shifted helix orientation (Figure S1A). Despite differences in their overall amino acid sequences, ASD1, ASD2, and ASD4 are predicted to engage a similar region on Flpp3 with subtle differences in the types of interface contacts. ASD1 and ASD4 both include an Asp residue that forms a polar interaction with Thr42, Phe43, and Arg102 of Flpp3, while ASD2 places a Trp at the same position. ASD2 and ASD4 also share an Arg at an adjacent site, while ASD1 features a Tyr at the same location. ASD3 adopts a shifted backbone orientation and uses a different set of interface residues (Figure S1A,B). Consistent with this, residue–residue contact maps (Figure S1C)—where we refer to the three helices of the ASD scaffold as H1, H2, and H3 (design‐specific residue ranges provided in Figure S1 caption)—show that ASD1/ASD2/ASD4 contact the residues ∼95–110 of Flpp3 predominantly via helices H2/H3, while ASD3 contacts the same Flpp3 patch via helices H1/H2, indicating a binder backbone shift. To assess any differences in the helix bundle packing, we calculated helix–helix crossing angles and internal helix–helix buried surface area (BSA) (H1–H2, H2–H3, H1–H3), finding that ASD1/ASD2/ASD4 share the same packing regime (H1–H2 dominant), whereas ASD3 shows greater H2–H3 burial (Figure S1D) consistent with the binder backbone/register shift seen in the contact maps. Sequence comparisons show moderate similarities among ASD1, ASD2, and ASD4 (25–40% pairwise sequence identity), while ASD3 is more divergent from other designs (20–25% pairwise identity) (Figure S2). Analysis of sequence similarity to the full design libraries shows that all validated binders are distinct with no close sequence relatives (Figure S3). The design model for the β‐site binder BSD1 also shows an extensive network of hydrophobic and polar interactions. Together, these results highlight the ability of computational design to generate high‐affinity binders against multiple sites of a selected target.

### Optimization of the β‐Site Binders

**Figure 4:**
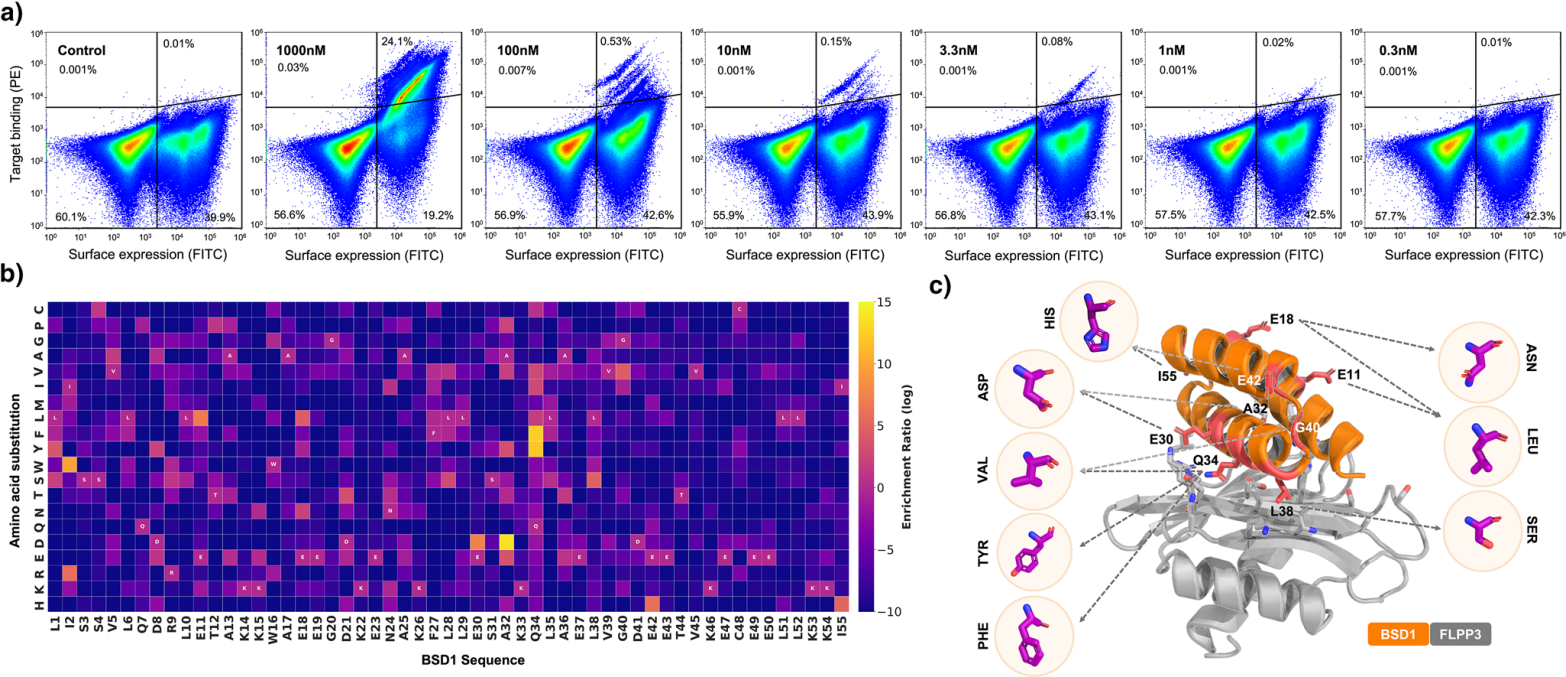
Site saturation mutagenesis optimization of β‐site binder BSD1. a) Fluorescence‐activated cell sorting plots show binding of BSD1 variants at decreasing concentrations of monomeric Flpp3 (1000 to 0.3 nM). b) Mutational enrichment heatmap of BSD1 SSM variants. The heatmap represents log‐transformed enrichment ratios of amino acid substitutions at each position of BSD1, derived from next‐generation sequencing (NGS) data across multiple FACS‐based binding sorts. Enrichment values were calculated by normalizing amino acid frequencies within the selected population relative to the naive library. Positions with high enrichment (yellow) correspond to beneficial mutations that enhance Flpp3 binding, whereas positions with low enrichment (purple) indicate substitutions that reduce binding. c) Structural mapping of enriched substitutions onto the BSD1. Selected residues on BSD1 are shown with side chains highlighted in coral, representing sites where substitutions were most strongly enriched in the YSD screen. Magenta callouts show example side chains predicted to improve binding affinity, based on enrichment data. These mutations primarily localize to the binder's interface with Flpp3, suggesting their role in stabilizing key interactions.

Since the β‐site binders generally demonstrated lower binding signal and affinities than α‐site binders during YSD and BLI experiments, we next set out to improve the affinity of the β‐site binder BSD1 using site saturation mutagenesis (SSM). A pooled library of 1046 BSD1 variants was generated by mutating each residue of BSD1 to 19 other canonical amino acids. Oligonucleotides encoding this variant library were commercially purchased, cloned into yeast cells, and screened for binding to Flpp3 using FACS as described earlier. In contrast to the original library of β‐site designs, which did not show a binding signal at 30 nM of Flpp3 (Figure 2b), the SSM‐optimized library showed a clear binding signal even during the sorting runs with 1 nM of monomeric Flpp3, indicating the presence of improved binders (Figure 4a).

**Figure 5:**
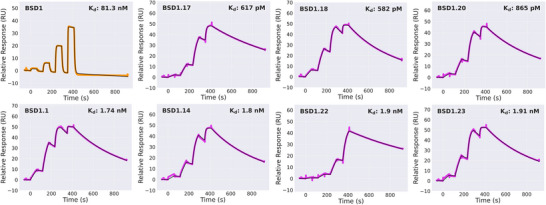
Determination of binding affinities for BSD1 and its selected variants using SPR. SPR sensorgrams from a four‐point single cycle kinetics experiment (5‐fold dilution, highest concentration ranging from 0.05 to 0.43 µM depending on the minibinder). Experimental data are shown in orange/magenta, and global fits are shown with black lines.

Sequencing data from the SSM library sorting supports the predicted binding mode of BSD1, with high conservation across positions making substantial interface contacts in the design model (Figure 4c). Additionally, the SSM data also suggested nine amino acid positions where mutations could potentially improve the binding affinity. Based on this data, we selected twelve single‐mutation variants, seven double‐mutation variants, and five triple‐mutation variants of BSD1 for further characterization (Table 1). All variants (BSD1.1–BSD1.24) were expressed in *E. coli*, purified using IMAC and SEC, and assessed for Flpp3 binding using surface plasmon resonance (SPR) (Figure 5 and Figure S8). Two variants (BSD1.21 and BSD1.24) did not express well and were not pursued further. The native design showed a K_D_ of 81 nM in SPR single‐cycle kinetics experiments (compared to 190 nM by BLI). 18 out of the 22 tested variants showed better *K_D_* than the original binder BSD1, with 13 variants yielding *K_D_* values less than 10 nM. Notably, single‐residue mutation of an interface‐facing Gln34 to Phe in BSD1.1 improved the binding affinity from 81 to 1.7 nM. Two double‐mutation variants (Q34F/I55H, and Q34F/L38S) and one triple‐mutation variant (Q34F/E11L/E30D) showed sub‐nanomolar binding affinities, with the two best variants, BSD1.17 and BSD1.18, displaying *K_D_* of 582 and 617 pM, respectively. Both BSD1.17 and BSD1.18 are still predicted to fold and bind similarly to BSD1 by AF3 (Figure S11). While 9 out of the 10 tested double or triple substitution variants yielded comparable or better activity than the best single substitution variant (Q34F), one double substitution variant (BSD1.15) demonstrated worse binding affinity than the individual Q34F and A32D variants. AF3 predictions indicate that the overall fold and binding mode are preserved in both the single and double mutants; however, the reduced affinity could be a result of subtle perturbations in local packing interactions or other incompatibility between these two mutations that are not correctly captured in our computational models. Notably, all variants with sub‐nanomolar binding affinity include the Q34F mutation, highlighting the importance of this particular interface mutation toward achieving improved binding affinity.

**Table 1:** Binding affinities of BSD1 variants against Flpp3.

Name	Substitution(s)	Binding affinity (nM)
BSD1	Original binder	81.30
BSD1.1	Q34F	1.74
BSD1.2	Q34Y	5.72
BSD1.3	Q34V	12.84
BSD1.4	E11L	9.50
BSD1.5	E18N	249.80
BSD1.6	E18L	8.72
BSD1.7	E30D	17.62
BSD1.8	A32D	8.24
BSD1.9	L38S	367.60
BSD1.10	G40V	59.40
BSD1.11	E42H	87.35
BSD1.12	I55H	31.46
BSD1.13	Q34F, E11L	1.67
BSD1.14	Q34F, E30D	1.80
BSD1.15	Q34F, A32D	125.10
BSD1.16	Q34F, E42H	2.73^a)^
BSD1.17	Q34F, L38S	0.62
BSD1.18	Q34F, I55H	0.58
BSD1.19	E11L, E30D	13.77
BSD1.20	Q34F, E11L, E30D	0.86
BSD1.21	Q34F, E11L, E42H	N/A
BSD1.22	Q34F, E11L, L38S	1.90
BSD1.23	Q34F, E30D, I55H	1.91
BSD1.24	E11L, E30D, E42H	N/A

Summary of single and combinatorial amino acid substitutions introduced into the parental minibinder BSD1. Binding affinities (in nM) were measured by SPR. Variants are sorted by name, and substitutions are listed relative to BSD1.

^a)^ *the global fit was not sufficiently high quality*

### Thermal Stability and Secondary Structure Characterization by Circular Dichroism

Next, we used circular dichroism (CD) to assess the thermal stability and obtain low‐resolution structural information for the designed minibinders. We measured the CD spectra for the two best α‐site designs (ASD1 and ASD2), the best β‐site design from the first round (BSD1), and the best variant of BSD1 after SSM maturation (BSD1.18). Both α‐site designs, ASD1 and ASD2, show a spectrum consistent with an all‐helical topology at 25 °C (Figure S9A, left panel). The thermal melt curves show that ASD1 and ASD2 unfold when heated to 95 °C, with melting temperatures of 55 and 65 °C, respectively (Figure S9A, right panel). However, both ASD1 and ASD2 refold to the original fold upon cooling to 25 °C (Figure S9A, left panel). Similar to α‐site binders, the β‐site binder BSD1 also shows spectra typical for an all‐helical topology at 25 °C, suggesting that it adopts the designed topology (Figure S9B, left panel). The minimal CD change observed for both β‐site binders during thermal melting experiments indicates that they remain folded across the tested temperature range (25–95 °C), suggesting a hyperstable structure with a melting temperature beyond 95 °C (Figure S9B, right panel). These data suggest that both BSD1 and the SSM‐optimized β‐site binder BSD1.18 are more thermostable than the α‐site binders. Overall, the α‐site and β‐site binders adopt the designed topology and refold to the designed fold even after thermal denaturation up to 95 °C and subsequent cooling.

### Structure Determination of Flpp3–ASD1 Complex by X‐ray Crystallography

**Figure 6:**
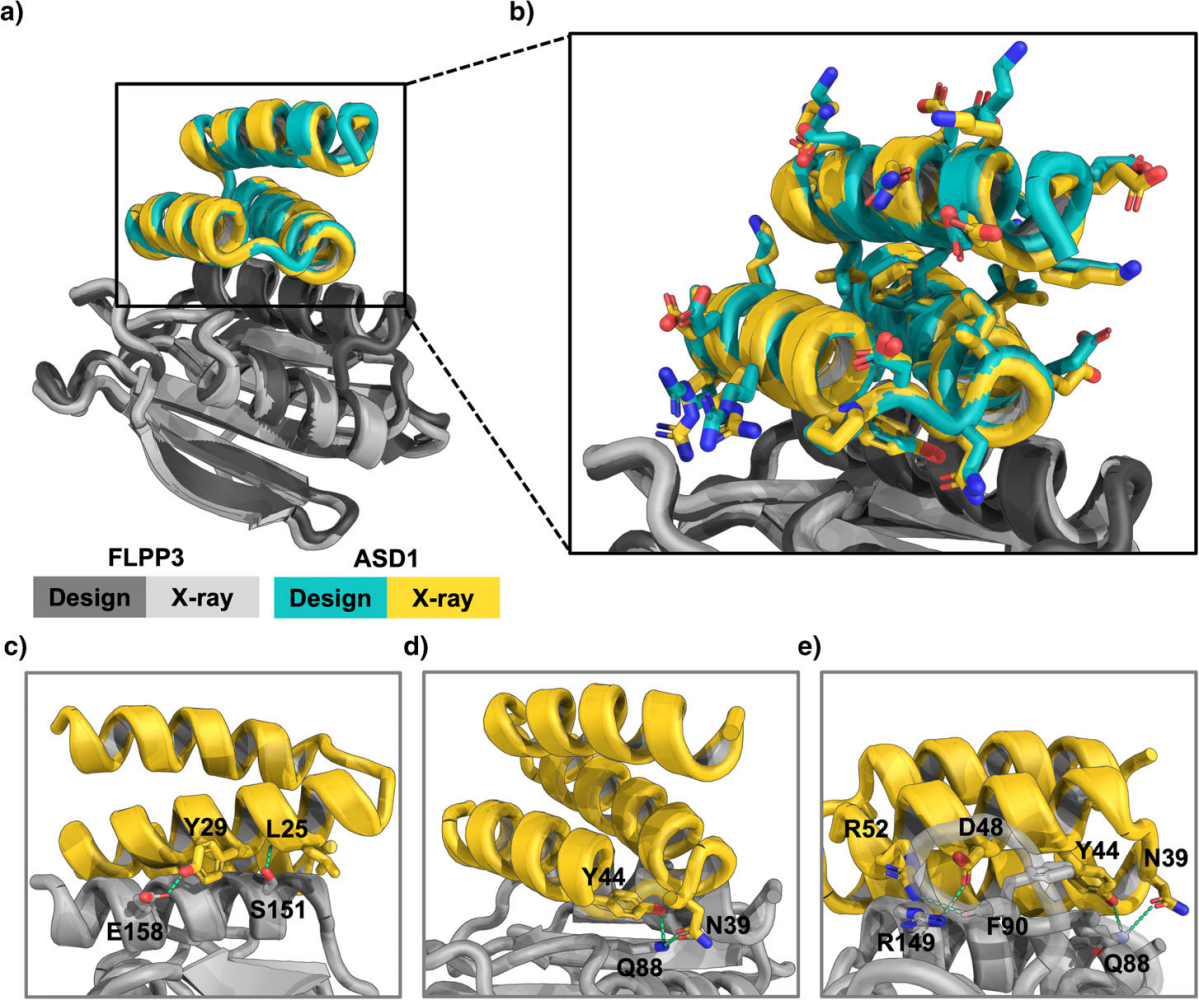
Crystal structure of the ASD1–Flpp3 complex confirms the designed binding mode. a) Overall structure of the ASD1–Flpp3 complex. The Flpp3 crystal structure is shown in light gray and ASD1 in yellow. The design models are overlaid in dark gray (Flpp3) and teal (ASD1). b) Zoomed‐in view of ASD1 design model and crystal structure with sidechains, showing close structural agreement. Cα RMSD for ASD1 is 0.9 Å when the structures are aligned by Flpp3 residues, and 0.4 Å when aligned by the binder only. c)–e) Key interactions between ASD1 and Flpp3, shown from different orientations of the interface. Key polar interactions between ASD1 and Flpp3 are shown as green dashed lines.

To confirm the three‐dimensional structure and binding mode of our designed minibinders, we pursued crystallization trials for several α‐site and β‐site designs in complex with Flpp3. Among these, only the highest‐affinity α‐site binder, ASD1, yielded crystals suitable for X‐ray diffraction. We determined the X‐ray crystal structure of the ASD1–Flpp3 complex at 2.37 Å resolution. The structure matches very closely with the design model of Flpp3‐bound ASD1, with a Cα RMSD of 0.9 Å when aligned by Flpp3 residues and 0.4 Å when comparing the miniprotein alone (Figure 6a,b). To our knowledge, this is the first high‐resolution structure of Flpp3 bound to a ligand. The crystal structure confirms that ASD1 engages the α‐site of Flpp3 through a network of hydrophobic and polar interactions, primarily mediated by the helices 2 and 3 of the miniprotein. The hydrophobic interactions at the core of the interface are mediated by ASD1 residues Leu25, Ala28, Leu33, Tyr44, Leu47, and Ala51, which pack against the complementary hydrophobic regions of the Flpp3 α‐site. Additionally, several polar interactions contribute to the binding interface. ASD1 residues Leu25 and Tyr29 interact with Flpp3 residues Ser151 and Glu158, respectively (Figure 6c). Tyr44 and Asn39 of ASD1 are also optimally positioned to form hydrogen bonds with Gln88 on Flpp3 (Figure 6d). The binding interface is further stabilized by additional polar contacts involving ASD1 residues Arg52, Asp48, Tyr44, and Asn39, which interact with Flpp3 residues Arg149, Phe90, and Gln88, respectively (Figure 6e). Notably, the sidechain rotamers of interface residues in the X‐ray crystal structure are nearly identical to those in the design model (Figure S10). Overall, the X‐ray structure of ASD1‐Flpp3 complex confirms the structure and binding mode of ASD1, highlighting the atomic accuracy provided by our computational design approach.

### Characterization of Flpp3 Surface Accessibility

To investigate the cellular location and orientation of Flpp3 in the outer membrane, we used flow cytometry to assess whether designed minibinders could label Flpp3 on the surface of intact *Francisella* cells. FITC‐conjugated ASD1 minibinder was incubated with *Francisella novicida* at concentrations ranging from 0–10 µM. We observed no significant change in cell labeling between wild‐type (WT) and Δ*flpp3* deletion strains at any of the tested concentrations (Figure S12B), suggesting that ASD1 does not bind Flpp3 on intact bacterial cells. To confirm if Flpp3 is expressed and potentially accessible on the surface of intact cells, we also generated a *F. novicida* strain expressing Flpp3 fused to a VSV‐G tag. However, staining with anti‐VSV‐G antibody followed by a FITC‐conjugated secondary antibody also showed no detectable staining compared to control strains (Figure S12A). Similar labeling studies with biotinylated β‐site binder BSD1 and streptavidin‐phycoerythrin (SAPE) at a range of concentrations (0, 1, 5, and 10 µM) also showed no significant difference in fluorescence signal between the WT and knockout (KO) control strains (Figure S12C). Western blot analysis confirmed the expression of Flpp3 in the bacterial strains used for flow cytometry experiments (Figure S13), indicating that the lack of binding signal in intact cells was not due to expression issues. These results suggest that Flpp3 is either not accessible on the cell surface or is oriented in a manner that prevents minibinder access to either of the two selected sites. The complex lipopolysaccharides (LPS) barrier and outer capsule of Francisella may prevent access of medium‐to‐large molecular weight molecules, such as miniproteins and antibodies.^[^ ^7^, ^27^ ^]^ Alternatively, Flpp3 may be localized to the inner leaflet of the outer membrane or sequestered within oligomeric complexes, as has been proposed for other Francisella lipoproteins,^[^ ^28^, ^29^ ^]^ thereby rendering the selected binding sites inaccessible in intact cells. Additional co‐localization studies using immunoprecipitation or fluorescence microscopy in the cells are required to assess the overall selectivity of the designed minibinders and to evaluate if Flpp3 is localized to the inner leaflet.

## Conclusion

The natural and deliberate spread of *F. tularensis* remains a significant concern because of its exceptionally high infectivity and low doses required to cause a severe infection. In this work, we used a combination of physics‐based and deep‐learning computational methods to design high‐affinity miniprotein binders targeting Flpp3, a key virulence factor of *F. tularensis*.

The computational approach described here overcomes several challenges in targeting Flpp3. Although the role of Flpp3 as a virulence factor is well established, structural and mechanistic understanding of Flpp3 and its interacting partners remains limited. The lack of Flpp3‐ligand complex structures and well‐defined binding pockets on Flpp3 surface make ligand discovery challenging. Despite this limited structural information, our computational methods enabled exploration of a vast conformational space to design and validate multiple miniprotein binders against two different sites of Flpp3. For α‐site, we identified four designed binders with nanomolar binding affinities, with the best binder displaying a K_D_ of 24 nM without any additional sequence optimization. While the initial binder for β‐site had a binding affinity of 81 nM, a round of site saturation mutagenesis identified multiple variants with binding affinities below 1 nM, including the best binder with a binding affinity of 580 pM. Circular dichroism spectroscopy confirmed that these binders fold into their designed all‐helical topology. X‐ray crystal structure of the best α‐site binder, ASD1, in a complex with Flpp3 confirms that ASD1 engages Flpp3 through the designed interface, with close agreement between the experimental structure and the design model. The close agreement between the X‐ray crystal structure and computational model of ASD1‐Flpp3 complex (Cɑ RMSD: 0.9 Å) highlights the remarkable accuracy of our design methods integrating deep learning and physics‐based methods and precision with which computationally designed miniproteins can be designed *de novo* to target difficult protein targets.

This work highlights the increased robustness, the ease of use, and considerable potential for computational approaches to custom design miniproteins with diverse shapes and sizes from scratch to bind selected protein targets, including bacterial virulence factors. The data generated here for Flpp3 binders, and their subsequent optimization should also serve as additional data points for further improving these design pipelines and in silico filtering strategies. The ability to computationally design protein binders with atomic level accuracy offers a powerful alternative to conventional library‐based screening methods or antibodies as it provides faster routes to affinity reagents with desired biophysical properties. The designed binders targeting different sites on Flpp3 also offer a means to precisely perturb its interactions with other proteins and explore the molecular mechanisms employed by *F. tularensis* during infection and immune evasion. While the flow cytometry experiments did not detect binder labeling on the surface of intact cells, these results suggest that Flpp3 may be positioned on the inner leaflet of the outer membrane or embedded within larger protein assemblies. In this context, the designed binders with their high specificity and affinity could serve as useful molecular tools to investigate Flpp3 localization, orientation, and structural environment in the native membrane using established tools like immunoprecipitation or fluorescence microscopy.

These miniproteins can further guide the development of small molecules and peptides with better access to the outer membrane proteins like Flpp3, penetration across the LPS and outer membrane, and superior drug‐like properties. Future research efforts will focus on optimizing the selected binders, leveraging them to explore the precise mechanistic roles of Flpp3 in infection and immune evasion, and evaluating their therapeutic potential.

### Author Contributions

G.G.A., B.H., J.D.M., and G.B. conceived and conducted the study. G.G.A., G.B., L.S.K., Y.W., and A.K.B wrote the manuscript. G.G.A. and B.H. performed yeast surface display screenings. L.S.K. performed the bacterial cell binding flow cytometry assays. D.K.V., N.R., and I.G. carried out yeast library transformations. A.L. and S.R.G. expressed and purified Flpp3. V.A. and G.G.A. expressed and purified the designed minibinders. A.L. and Y.F.B. prepared plasmids for Flpp3 expression. Y.W. designed and constructed the ∆*flpp3* and the *flpp3*–VSV‐G strains and performed the western blot experiment to confirm Flpp3 expression in the relevant strains. S.R. contributed to initial SPR assay development and performed preliminary experiments. G.G.A. conducted subsequent SPR experiments and data analysis. X.L. verified minibinder integrity via mass spectrometry. A.K.B. and A.K. determined the X‐ray crystal structures of the designed minibinders bound to Flpp3. D.B., J.D.M., J.J.W., and G.B. supervised the project. All authors contributed to data interpretation and manuscript revision. G.G.A. and B.H. agree to be considered co‐first authors and that their names may be reordered for personal or professional purposes.

## Conflict of Interests

D.B. and G.B. are co–founders, advisors, and shareholders of Vilya Therapeutics, a biotech company.

## Supporting information



Supplementary Information

Supplementary Information

## Data Availability

The data that support the findings of this study are available in the Supporting Information of this article.
