# Supplementary material for: De Novo Design of High‐Affinity Miniprotein Binders Targeting Francisella Tularensis Virulence Factor
Source: Angew Chem Int Ed Engl. 2025 Oct 21;64(52):e202516058. doi: 10.1002/anie.202516058 (PMC12723454; doi:10.1002/anie.202516058)
Supplement: Supplementary file 1 — Supplementary Information [file ANIE-64-e202516058-s001.docx]

**De Novo Design of High-affinity Miniprotein Binders Targeting *Francisella tularensis* Virulence Factor**

**Authors:** Gizem Gokce-Alpkilic^1,2,3*^, Buwei Huang^3,4,5,6*^, Andi Liu^7,8^, Lieselotte S.M. Kreuk^7^, Yaxi Wang^7^, Victor Adebomi^2,3^, Yensi Flores Bueso^3,9^, Asim K. Bera^3^, Alex Kang^3^, Stacey R. Gerben^3^, Stephen Rettie^2,3,10^, Dionne K. Vafeados^3^, Nicole Roullier^3^, Inna Goreshnik^3^, Xinting Li^3^, David Baker^3,4,11^, Joshua J. Woodward^7^, Joseph D. Mougous^7,11,12,^**^‡^**, Gaurav Bhardwaj^1,2,3,^**^‡^**

**Author Affiliations**

1. Molecular Engineering and Sciences Institute, University of Washington, Seattle, WA, USA
2. Department of Medicinal Chemistry, University of Washington, Seattle, WA, USA
3. Institute for Protein Design, University of Washington, Seattle, WA, USA
4. Department of Biochemistry, University of Washington, Seattle, WA, USA
5. Department of Bioengineering, University of Washington, Seattle, WA, USA
6. Current address: Xaira Therapeutics, Seattle, WA, USA
7. Department of Microbiology, University of Washington, Seattle, WA, USA
8. Current address: Sound Biologics, Bothell, WA, USA
9. Cancer Research @UCC, University College Cork, Cork, Ireland
10. Molecular and Cellular Biology Program, University of Washington, Seattle, WA, USA
11. Howard Hughes Medical Institute, University of Washington, Seattle, WA, USA
12. Microbial Interactions and Microbiome Center, University of Washington, Seattle, WA, USA

*These authors contributed equally.

**^‡^**Corresponding authors: Gaurav Bhardwaj (gauravb@uw.edu), Joseph Mougous (joseph.mougous@yale.edu)

**SUPPORTING INFORMATION**

**METHODS**

***De novo* Computational Design**

Miniprotein binders against Flpp3 were designed using a multi-step computational pipeline integrating physics-based docking and deep-learning-based sequence optimization. The soluble domain structure of Flpp3 (PDB ID: 6PNY) was used as the design target, and two distinct regions on it were selected to guide the binder design calculations: the α-site (α-helical, electropositive face) and the β-site (β-sheet, electronegative face). A hierarchical docking approach was applied to identify scaffold backbones with high binding potential. First, disembodied amino acid side chains were docked against the selected target regions using Rosetta RIFGen^[[13]](https://paperpile.com/c/NMKabq/t2HPc)^ approach described previously. Following general approaches outlined in prior RIF-based binder design work[^[13]^](https://paperpile.com/c/3hj1pe/SM1rV), RIFGen was used to build a lookup table of sidechain–target interactions, allowing rapid scoring of potential scaffold placements. Next, PatchDock[^17^](https://paperpile.com/c/3hj1pe/mU5Gl) was used to perform a global search for shape complementarity between scaffolds and the Flpp3 surface, identifying placements where overall geometry of the pre-enumerated scaffolds was complementary to the selected Flpp3 surface. These PatchDock placements were then refined with RIFDock^[[13]](https://paperpile.com/c/3hj1pe/SM1rV)^, which uses the precomputed rotamer interaction fields to optimize sidechain packing and rigid-body orientation, thereby improving the quality of the docked models. From this hierarchical docking strategy, we generated 500,000 docked conformations that were carried forward into sequence design. Next, ProteinMPNN^[[](https://paperpile.com/c/3hj1pe/SM1rV)^[^18^](https://paperpile.com/c/3hj1pe/mWbn9)[^]^](https://paperpile.com/c/3hj1pe/SM1rV) was applied to redesign the amino acid sequences of the selected scaffold backbones. ProteinMPNN does not directly incorporate RIFGen information, instead, it improves chemical and structural complementarity by optimizing sequences for backbones that have already been positioned and refined through RIFDock (unlike Rosetta FastDesign where RIFGen amino acids are kept fixed). Two iterative rounds of ProteinMPNN were performed, each followed by Rosetta FastRelax^[[](https://paperpile.com/c/3hj1pe/SM1rV)^[^26^](https://paperpile.com/c/3hj1pe/572ao)[^]^](https://paperpile.com/c/3hj1pe/SM1rV) to allow limited backbone relaxation and increase sequence diversity. Designed sequences were filtered using Rosetta interface energy metrics (ddG < -40 kcal/mol for α-site, < -35 kcal/mol for β-site) and structural confidence scores from AlphaFold2 (pLDDT > 90 for α-site, > 80 for β-site; iPAE < 6.0)[^[15]^](https://paperpile.com/c/NMKabq/71oOQ). AlphaFold2 predictions were run using the AF2 model_1_ptm weights, which provides both pLDDT and iPAE confidence metrics. This model is monomer-trained, rather than multimer-trained, and thus does not introduce explicit biases toward complex stabilization. To remove aggregation-prone sequences, designs were further filtered using spatial aggregation propensity (SAP) scores (<30 for α-site, <35 for β-site)[^[13]^](https://paperpile.com/c/NMKabq/t2HPc). The final selection yielded 15,000 α-site designs and 8,817 β-site designs, which were experimentally validated using yeast surface display and biochemical assays.

**Yeast Surface Display and Fluorescence-Activated Cell Sorting**

We used yeast surface display (YSD) to screen and enrich miniprotein binders against Flpp3. Saccharomyces cerevisiae EBY100 cells were transformed with synthetic genes encoding the designed minibinders using a pETCON3 expression vector, enabling surface display via the Aga2p system[^[30]^](https://paperpile.com/c/NMKabq/fUm6E). Transformed yeast cells were cultured in C-Trp-Ura medium supplemented with 2% (w/v) glucose at 30°C, shaking at 220 rpm. For induction, cells were centrifuged, resuspended in SGCAA medium supplemented with 0.2% (w/v) glucose, and incubated at 30°C for 16–18 hours. The screening process consisted of four sequential fluorescence-activated cell sorting (FACS) rounds. The first round (called expression sorting) was performed without Flpp3 to isolate yeast cells successfully expressing minibinders on the surface. In the second round (initial avidity sorting), yeast cells were incubated with 1 μM Flpp3, anti-c-Myc fluorescein isothiocyanate (FITC) and streptavidin-phycoerythrin (SAPE) to enrich for binders. The third round, enrichment sorting, was conducted again at 1 μM Flpp3 to further increase the population of Flpp3-binding cells. In the final round (titration sorting), yeast cells were incubated with decreasing concentrations of Flpp3 (1,000 nM, 100 nM, 10 nM, 1 nM for the α-site library; 1,000 nM, 300 nM, 100 nM, 30 nM for the β-site library) to select for higher-affinity binders. FACS was performed using the Sony SH800 Cell Sorter, with gating thresholds established using a negative control population (yeast cells incubated without Flpp3) to define background fluorescence. Yeast populations were sorted based on this gated threshold, ensuring that only Flpp3-binding cells above background fluorescence were collected. Data were plotted and analyzed using FlowJo v10[^[31]^](https://paperpile.com/c/NMKabq/qGdxc). Sorted yeast populations were lysed using Zymolyase-Yeast Lytic Enzyme from the Zymoprep Yeast Plasmid Miniprep I kit, and enriched sequences were recovered for next-generation sequencing (NGS) to evaluate sequence enrichment across sorting rounds. NGS was performed following the protocol described by Cao et al. (2022)[^[13]^](https://paperpile.com/c/NMKabq/t2HPc).

**Flpp3 Expression and Purification**

The gene encoding Flpp3, containing an N-terminal His6-Avi tag, was cloned into the pET-28a(+) expression vector and transformed into chemically competent E. coli BL21 (DE3) cells (NEB C2527I) following the recommended protocols. The plasmid contains a kanamycin resistance gene for selection. A single colony was inoculated into 3–4 mL of LB medium supplemented with kanamycin (50 µg/mL) and grown overnight at 37°C with shaking at 200 rpm. The overnight culture was then diluted into 1 L of 2XYT medium in 2 L baffled flasks and incubated at 37°C until reaching an OD600 of approximately 0.4. The incubation temperature was then reduced to 18°C, and after 30 min of acclimatization, protein expression was induced by adding isopropyl β-D-1-thiogalactopyranoside (IPTG) to a final concentration of 0.3 mM. The culture was incubated for an additional 16–20 hours at 18°C with continuous shaking. Cells were harvested by centrifugation at 9,000 × g for 20–30 min at 4°C, and the resulting cell pellet was either flash-frozen in liquid nitrogen for storage at -80°C or immediately processed for purification. For protein purification, the cell pellet was resuspended in 40 mL of lysis buffer per liter of culture (50 mM Tris-HCl, pH 7.5, 500 mM NaCl, 10% glycerol, 5 mM imidazole) supplemented with lysozyme (0.5 mg/mL), protease inhibitors (Pierce™ Protease Inhibitor, A32963), and Benzonase nuclease (Thermo Scientific, E1014) following manufacturer’s recommendations. The resuspended cells were lysed by sonication (30 s pulses at 50% amplitude, repeated for 4–5 cycles with 5–10 min cooling intervals on ice between rounds). The lysate was cleared by centrifugation at 17,000 × g for 30 min at 4°C, and the supernatant was transferred to a fresh tube. Purification was performed using immobilized metal affinity chromatography (IMAC) on a 1 mL HisTrap column (Cytiva) connected to an ÄKTA FPLC system. The column was equilibrated with Ni-Buffer A (50 mM Tris-HCl, pH 7.5, 500 mM NaCl, 10% glycerol, 5 mM imidazole), and the clarified lysate was loaded onto the column at a flow rate of 1 mL/min. The column was washed extensively with Ni-Buffer A to remove non-specifically bound proteins, and the target protein was eluted using an imidazole gradient (0–100% Ni-Buffer B containing 50 mM Tris-HCl, pH 7.5, 500 mM NaCl, 10% glycerol, and 500 mM imidazole). The eluted fractions were analyzed using SDS-PAGE, and those containing Flpp3 were pooled and concentrated using Amicon Ultra centrifugal filters (3 kDa MWCO) by centrifugation at 2,000–3,000 × g at 4°C. For further purification and buffer exchange, the protein was subjected to size exclusion chromatography (SEC) using a HiLoad 16/600 Superdex 200 column pre-equilibrated with sizing buffer (50 mM Tris-HCl, pH 7.5, 500 mM NaCl, 10% glycerol). The protein was loaded manually using a 1-2 mL sample loop and eluted at a flow rate of 0.5 mL/min. Fractions corresponding to Flpp3 were identified by A280 absorbance, pooled, and concentrated as described above. Protein concentration was determined using a NanoDrop spectrophotometer (Thermo Scientific) based on its predicted extinction coefficient. The purified protein was aliquoted, snap-frozen in liquid nitrogen, and stored at -80°C until further use.

**Miniprotein Expression and Purification**

Minibinders were expressed in *E. coli* using a small-scale expression system in a 96-well deep-well plate format. Genes encoding the designed minibinders were synthesized by Integrated DNA Technologies (IDT) and cloned into the LM0627 (Addgene 191551) BVN2 expression vector with a C-terminal SNAC[^[32]^](https://paperpile.com/c/NMKabq/7Cgdr) and 6×His tag for affinity purification. The plasmids were transformed into E. coli BL21(DE3) competent cells (New England Biolabs). Transformed cells were grown overnight in LB medium supplemented with kanamycin (50 µg/mL) at 37°C with shaking at 900 rpm in a deep-well plate incubator. The following day, overnight cultures were diluted 1:20 into fresh Terrific Broth (TB2) with autoinduction media supplemented with kanamycin (50 µg/mL) and 0.5% glycerol. Cultures were grown at 37°C with shaking for 18 h. Cells were harvested by centrifugation at 3,220 × g for 10 minutes at 4°C, and the resulting cell pellets were resuspended in 200 µL of lysis buffer per well. The lysis buffer consisted of BPER supplemented with 0.1 mg/ml lysozyme, 10 µg/ml DNase I and 1 mM PMSF. Lysis was carried out by incubation at room temperature with shaking for 30 minutes. The lysates were clarified by centrifugation at 4000 × g for 10 minutes at 4°C, and the supernatants were collected for purification. Minibinders were purified using Ni-NTA resin (Qiagen) in a high-throughput format. The resin was equilibrated with the wash buffer (50 mM Tris-HCl, 300 mM NaCl, 25 mM imidazole, pH 8.0) before being added to the clarified lysates. Binding was performed at room temperature for 1 hour, after which the resin was washed three times with wash buffer to remove unbound proteins. Minibinders were eluted in elution buffer (50 mM Tris-HCl, 300 mM NaCl, 500 mM imidazole, pH 8.0), and protein-containing fractions were collected. To further purify the minibinders, the eluates from Ni-NTA purification were subjected to size-exclusion chromatography (SEC) using a Superdex 75 Increase 10/300 GL column (Cytiva) equilibrated with SEC buffer (50 mM Tris-HCl, 150 mM NaCl, pH 8.0). Fractions corresponding to the expected molecular weight were collected and analyzed. Protein concentrations were determined using a NanoDrop spectrophotometer (Thermo Scientific) by measuring absorbance at 280 nm.

**Binding Affinity Determination Using Biolayer Interferometry and Surface Plasmon Resonance**

Binding affinities of the miniprotein hits identified from the yeast display screening were determined using biolayer interferometry using an Octet RED96 instrument (ForteBio). Streptavidin-coated biosensors (ForteBio) were first incubated with biotinylated Flpp3 at a concentration of 50–100 nM in BLI assay buffer (10 mM HEPES, 150 mM NaCl, 3 mM EDTA, 0.05% surfactant P20, and 1% BSA). Following target immobilization, biosensors were transferred to buffer-only wells to establish a baseline signal before being exposed to varying concentrations of minibinders to monitor the association phase. Next, the biosensors were returned to buffer-only wells for the dissociation phase to measure the off-rate (k_off_). The kinetic parameters, including association rate constant (k_on_), dissociation rate constant (k_off_), and equilibrium dissociation constant (K_D_), were determined using Octet Data Analysis software by fitting the data to a 1:1 binding model.

SPR experiments were conducted using a Cytiva Biacore 8K system with HBS-EP+ buffer (Cytiva) as the running buffer. Biotinylated Flpp3 was immobilized on a streptavidin-coated sensor chip using the Biotin Capture Kit (Cytiva). Prior to analyte injections, a capture test was performed to determine the optimal Flpp3 loading concentration, ensuring an appropriate surface density relative to the minibinders based on their molecular weight ratios. Binding interactions were assessed using single-cycle kinetics (SCK) at a flow rate of 30 µl/min. For the initial screening, minibinders were injected in a 4-point, 10-fold dilution series, with an association phase of 60 seconds followed by a 120-second dissociation phase. Based on the estimated K_D_ from the screening, a finer kinetic analysis was performed using a 4-point, 5-fold dilution series, extending the dissociation time to 500 seconds for improved resolution of slower off-rates. Sensorgrams were processed using Biacore Insight Evaluation Software, with double referencing applied. Binding kinetics were analyzed using a 1:1 binding kinetics fit model.

**Cell Surface Labeling**

For bacterial flow cytometry, *F. novicida* U112 cells were cultured overnight in tryptic soy broth supplemented with 0.1% (w/v) cysteine (Research Products International C81020) (TSB-C) from a single colony at 37 ℃ with shaking. On the day of the experiment, bacteria were diluted 1:50 in TSB-C and subcultured to mid-log phase (OD600nm~0.5). Bacteria were washed with sterile-filtered PBS with 1% bovine serum albumin (BSA; Research Products International A30075) and resuspended at approximately 1x10^7^ bacteria/mL. Minibinder was diluted in sterile PBS at various concentrations, and 25 µl of this solution was mixed with 25 µL diluted bacteria in a v-bottom plate. Cells were stained with minibinder for 1 hr at room temperature. For binding experiments using biotinylated minibinders, secondary staining of cells was performed with streptavidin-PE (Thermo Fisher 12-4317-87). Primary antibody staining of VSV-G tagged FLPP3 bacterial strains was performed with rabbit anti-VSV-G (Sigma Aldrich V4888), for 30 min at 4 ℃. Cells were washed and stained with fluorophore-labeled secondary antibody (goat anti-rabbit-488; Invitrogen A-11008). Cells were washed and fixed in 4% paraformaldehyde (PFA). After washing, bacteria were resuspended in PBS and analyzed by FACS. Data were acquired on an LSR II (BD Biosciences).

**Bacterial Strains and Growth Conditions**

Bacterial strains used in this study include *F. tularensis* subspecies *novicida* U112 (*F. novicida*, gift from Colin Manoil, University of Washington, Seattle, WA), *Escherichia coli* strain DH5α (*E. coli* DH5α, Thermo Fisher Scientific), and *E. coli* BL21 (DE3). *F. novicida* strains were grown aerobically at 37 °C in tryptic soy broth or agar supplemented with 0.1% (w/v) cysteine (TSBC or TSAC). For selection, kanamycin was used at the following concentrations: 15 μg/mL (*F. novicida*) or 50 μg/mL (*E. coli*). *F. novicida* strains were stored in TSBC supplemented with 20% (v/v) glycerol at -80 °C. *E. coli* strains were stored in LB supplemented with 15% (v/v) glycerol at -80°C.

**Strain and Plasmid Construction**

*F. novicida* ∆*flpp3* and *flpp3*–VSV-G strains were generated via allelic exchange as described previously[^[33–35]^](https://paperpile.com/c/NMKabq/N19RP+qf5Yd+UrRI7). Briefly, sequences containing 900 bp flanking the site of deletion or insertion were amplified by PCR and cloned into the BamHI and PstI sites of the vector pEX18-pheS-km using Gibson assembly[^[33]^](https://paperpile.com/c/NMKabq/N19RP). Naturally competent *F. novicida* was prepared by back-diluting overnight cultures in TSBC, growing for 3 hrs at 37°C with shaking, harvesting by centrifugation, and resuspending in *Francisella* transformation buffer[^[34]^](https://paperpile.com/c/NMKabq/qf5Yd). pEX18-pheS-km-based deletion or insertion plasmid was added to freshly prepared *F. novicida* competent cells. Bacterial suspensions were then incubated at 37 °C with shaking for 30 min, followed by addition of TSBC and an additional 3 hrs of incubation. Transformants were selected by plating on TSAC with kanamycin. The resulting merodiploids were grown overnight in non-selective TSBC, diluted into Chamberlain’s defined medium[^[36]^](https://paperpile.com/c/NMKabq/B2hQ3) containing 0.1% p-chlorophenylalanine (w/v) and allowed to grow to stationary phase. Cultures were then streaked onto TSAC, colonies were patched onto TSAC with and without kanamycin, and kanamycin-sensitive colonies were screened for mutations by colony PCR. Primers used in plasmid and strain construction can be found in Table S1.

**Flpp3–VSV-G Expression Analysis**

To analyze the expression of Flpp3, *F. novicida* wild-type and *flpp3*–VSV-G strains were grown overnight in TSBC at 37 °C with shaking, back-diluted the next morning, grown to OD_600_~0.9 and the equivalent of 1 ml culture at OD600=1 was collected for each strain by centrifugation. Cell pellets were resuspended in an equal volume of 1x Laemmli buffer[^[37]^](https://paperpile.com/c/NMKabq/orgYx) and heated at 95 °C. Proteins in each sample were separated by SDS-PAGE and analyzed by western blotting as described previously[^[35]^](https://paperpile.com/c/NMKabq/UrRI7).

**Antibodies**

Anti-VSV-G antibody produced in rabbit, Sigma V4888.

Anti-rabbit IgG (whole molecule)–peroxidase antibody produced in goat, Sigma A6154

**X-ray Crystallography for Flpp3-ASD1 complex**

All crystallization experiments were conducted using the sitting-drop vapor-diffusion method. Crystallization trials were set up in 200 nL drops using the 96-well plate format at 20˚C. Crystallization plates were set up using a Mosquito LCP from SPT Labtech, then imaged using UVEX microscopes and UVEX PS-256 from JAN Scientific. Diffraction quality crystals formed in 0.1 M Bis-Tris Propane pH 6.5, 0.2 M Sodium iodide, 20% (w/v) PEG 3350 and 10% (v/v) Ethylene glycol for the Flpp3-ASD1 complex. Diffraction data were collected at the National Synchrotron Light Source II beamline FMX (17-ID-2). X-ray intensities and data reduction were evaluated and integrated using XDS[^[38]^](https://paperpile.com/c/NMKabq/KK3x4) and merged/scaled using Pointless/Aimless in the CCP4 program suite[^[39]^](https://paperpile.com/c/NMKabq/YGfOR). Structure determination and refinement starting phases were obtained by molecular replacement using Phaser[^[40]^](https://paperpile.com/c/NMKabq/UPglJ) using the designed model for the binder and 6PNY for the Flpp3 structure. Following molecular replacement, the models were improved using phenix.autobuild^[[41]](https://paperpile.com/c/NMKabq/pEK8d)^; efforts were made to reduce model bias by setting rebuild-in-place to false, and using simulated annealing and prime-and-switch phasing. Structures were refined in Phenix[^[41]^](https://paperpile.com/c/NMKabq/pEK8d). Model building was performed using COOT[^[42]^](https://paperpile.com/c/NMKabq/dJQDB). The final model was evaluated using MolProbity^[[43]](https://paperpile.com/c/NMKabq/3cymm)^. Data collection and refinement statistics are recorded in Table S2. Data deposition, atomic coordinates, and structure factors reported in this paper have been deposited in the Protein Data Bank (PDB), http://www.rcsb.org with accession code 9NLT.

**Mass Spectrometry**

To confirm the molecular mass of each miniprotein and expressed proteins, intact mass spectra were obtained via reverse-phase LC/MS on an Agilent G6230B TOF using an AdvanceBio RP-Desalting column (Figure S7).

**Plotting and Figures**

Plots in this manuscript were generated using matplotlib[^[44]^](https://paperpile.com/c/NMKabq/JLHOV), seaborn[^[45]^](https://paperpile.com/c/NMKabq/UCfXR), FlowJo (v10), or GraphPad Prism (v10.4.1). Figures were created using PowerPoint v16.97.2 and BioRender. Protein structures were generated using PyMOL (version 2.5.2).

**SUPPORTING FIGURES**

**
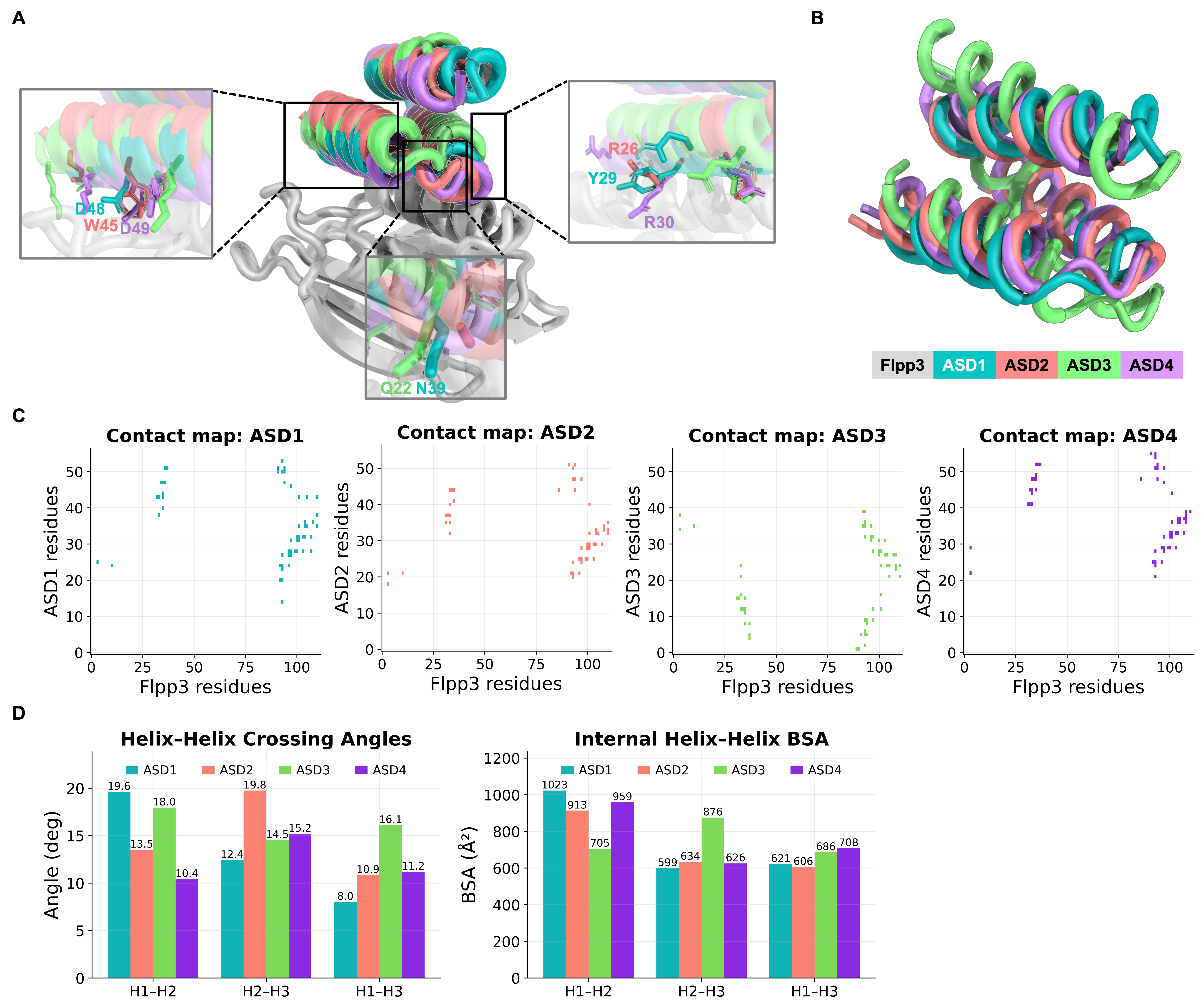
**

**Figure S1: Structural comparison of computationally designed α-site binders (ASD1–4).**

(A) Design models of ASD1 (teal), ASD2 (salmon), ASD3 (green), and ASD4 (purple) are shown superimposed in their binding poses on Flpp3 (gray). ASD side chains that form direct contacts to Flpp3 are shown as sticks (insets highlight representative regions). (B) Structural alignment of ASD1–4 based on Cα RMSD highlights differences in backbone geometry independent of Flpp3 binding context. Alignments were performed in PyMOL using backbone (Cα) atoms only, without using target as reference. (C) Residue–residue contact maps for ASD1–4 (binary contacts defined as any non-hydrogen atom pair within 4.5 Å). Helix definitions by residue numbers: ASD1 H1 = 1–18, H2 = 19–39, H3 = 40–54; ASD2 H1 = 1–16, H2 = 17–35, H3 = 36–53; ASD3 H1 = 1–21, H2 = 22–40, H3 = 41–56; ASD4 H1 = 1–19, H2 = 20–39, H3 = 40–57. ​​(D) Helix-packing metrics. Helix–helix crossing angles and internal helix–helix buried surface areas (BSA) for H1–H2, H2–H3, and H1–H3.


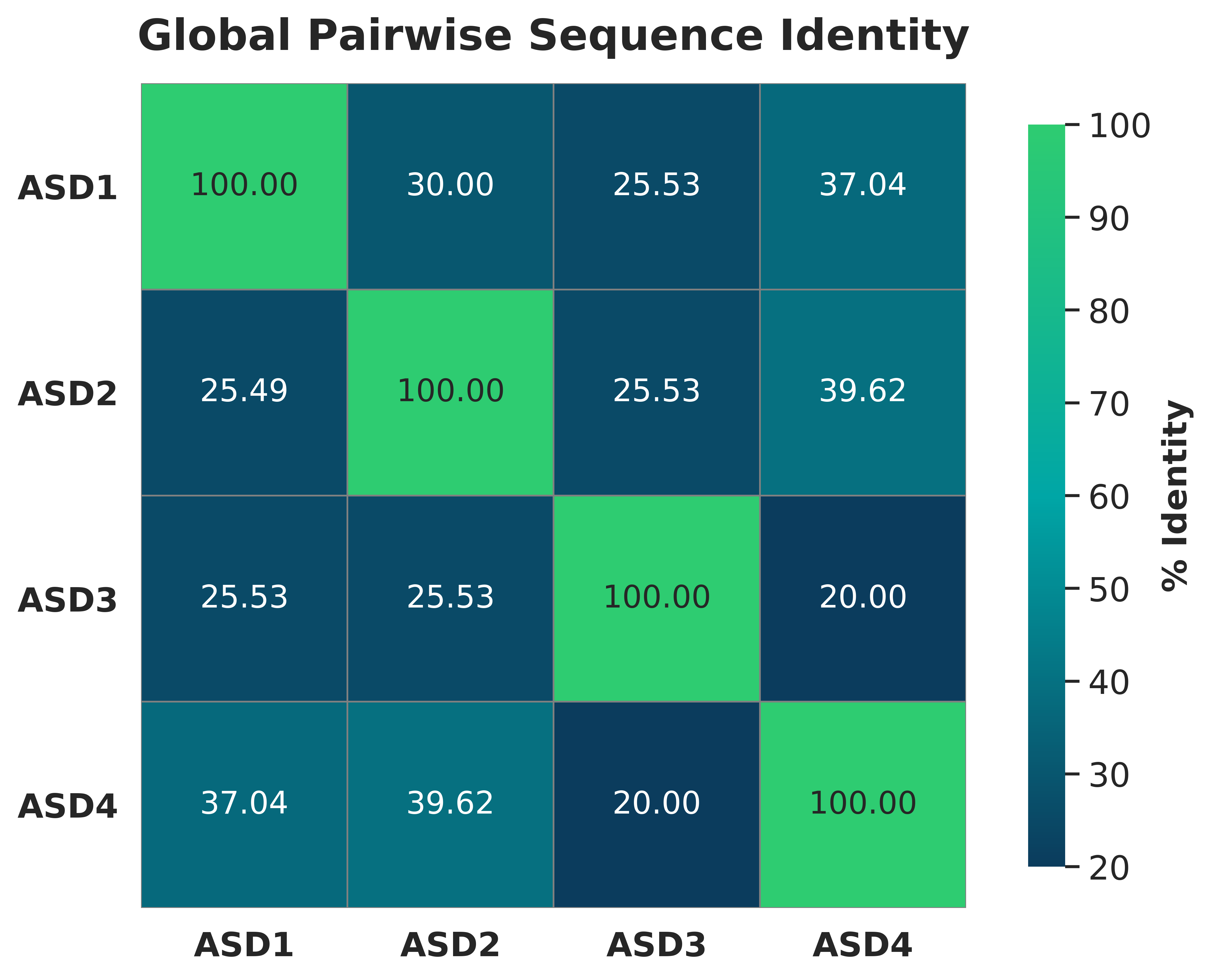


**Figure S2: Global pairwise sequence identity among α-site binders.**

Pairwise sequence identity heatmap for ASD1-ASD4 minibinders, calculated using global sequence alignment with the BLOSUM62 substitution matrix. Percent identities were computed based on aligned residues using the Needleman-Wunsch algorithm[^[46]^](https://paperpile.com/c/NMKabq/ZrFYA), with a gap opening penalty of –10 and a gap extension penalty of –1. Green indicates higher sequence identity, while darker blue corresponds to lower identity.


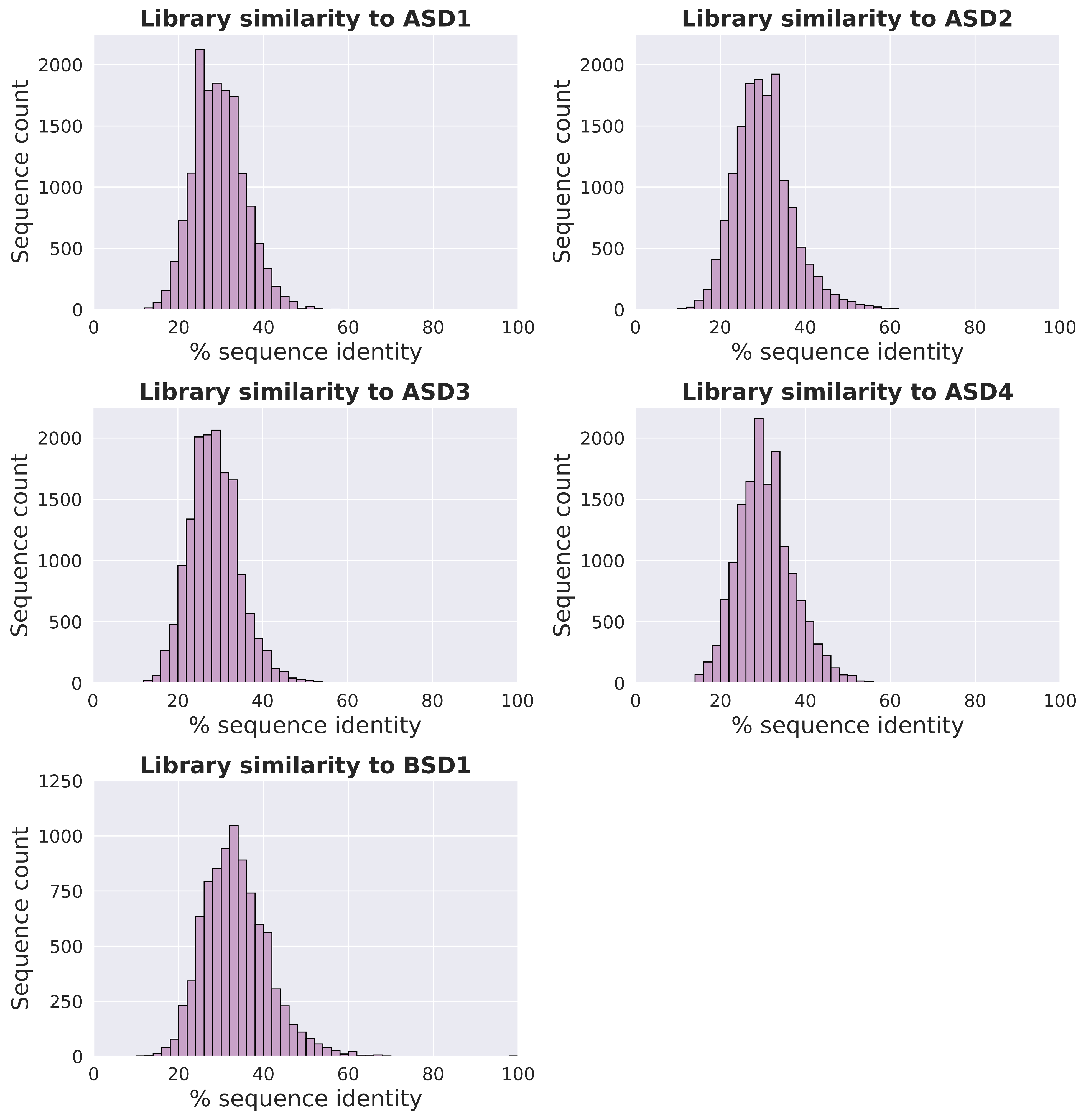


**Figure S3: Sequence similarity of validated binders to their respective design libraries.**

Histograms showing the distribution of global pairwise sequence identity between each validated binder (ASD1–ASD4 for the α-site library and BSD1 for the β-site library) and all other sequences in its corresponding design library. Percent identities were computed using global alignment with the BLOSUM62 substitution matrix and the Needleman–Wunsch algorithm, with a gap opening penalty of –10 and a gap extension penalty of –1.


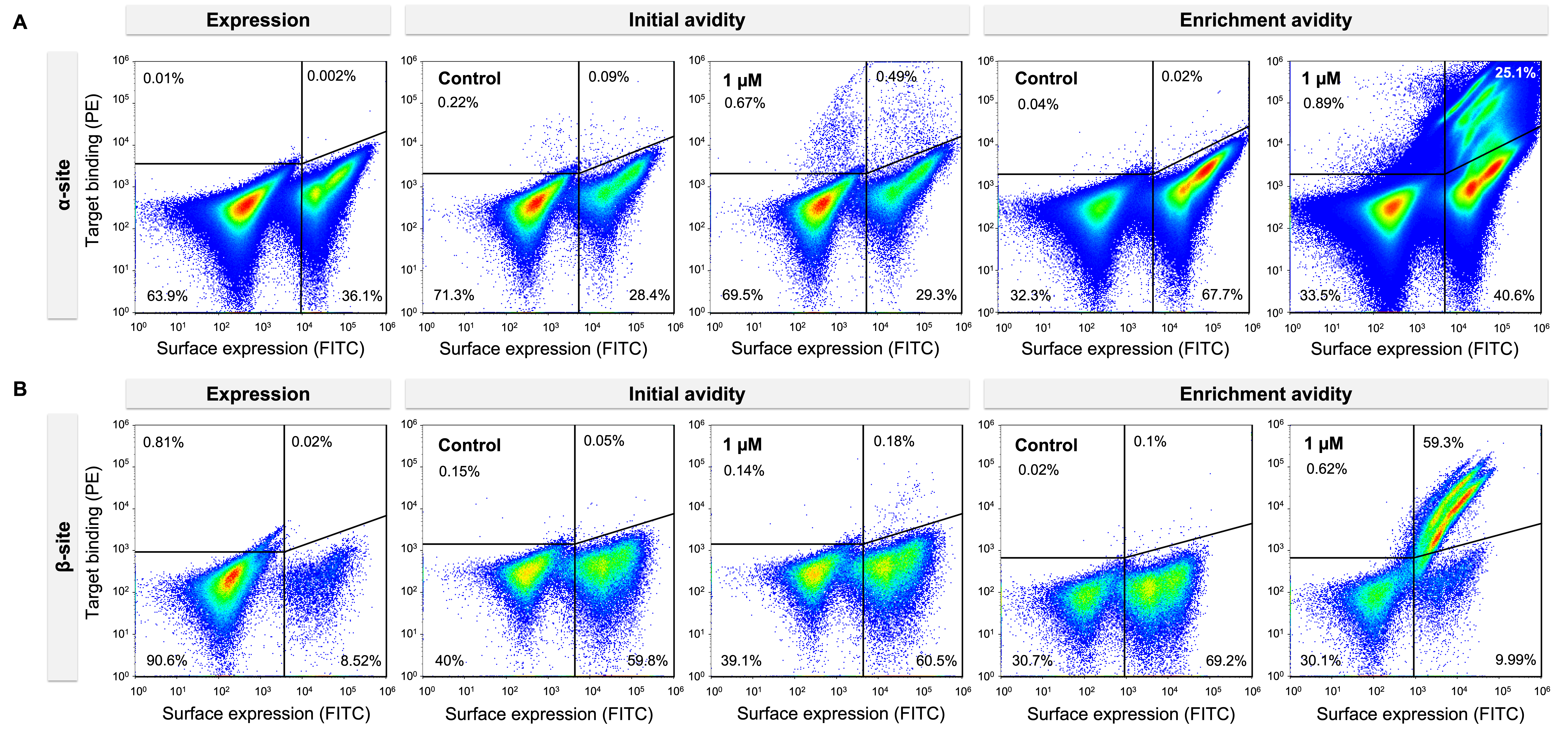


**Figure S4: Yeast surface display screening of α-site and β-site libraries against Flpp3 through expression and avidity-based enrichment.**

(A) Binding profiles of the α-site library during expression, initial avidity, and enrichment avidity sorts. A distinct population of Flpp3-binding cells is observed in the enrichment avidity sort at 1 µM. (B) Binding profiles of the β-site library under the same conditions. A clear binding population is also observed in the Enrichment avidity sort at 1 µM.


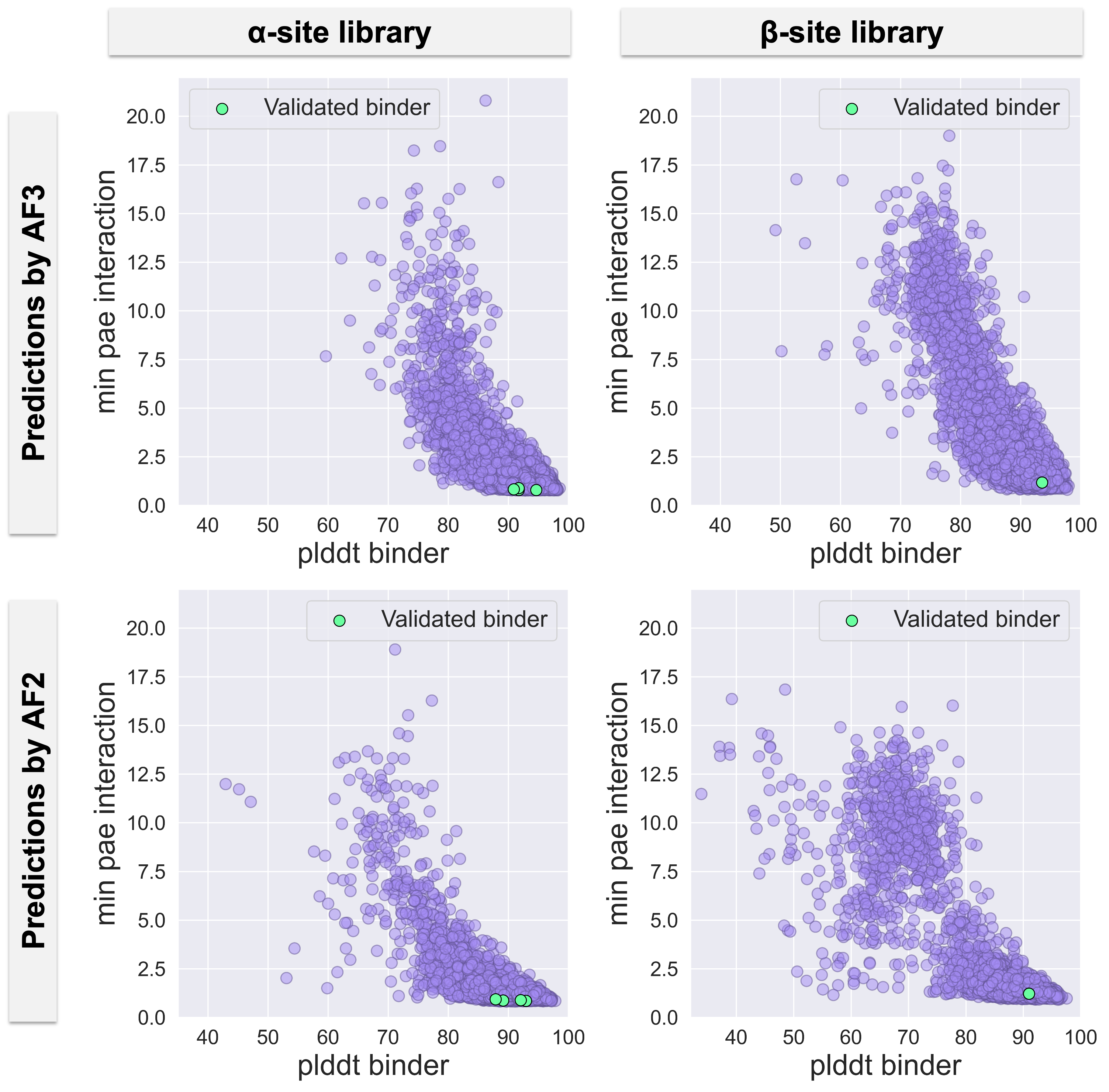


**Figure S5: Predicted confidence metrics for all designs compared to validated binders.**

Scatter plots of predicted binder pLDDT versus minimum interface PAE for all α-site (left) and β-site (right) library designs, as calculated by AF3 (top) and AF2 with multimer weights (bottom). Each purple point represents one design. Experimentally validated binders (ASD1–ASD4 and BSD1) are highlighted in green.


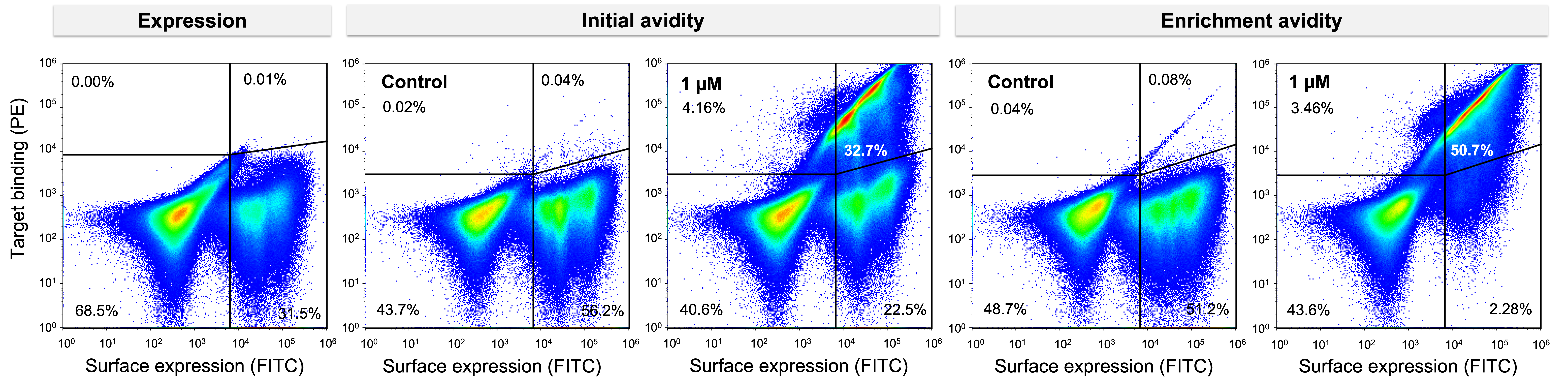


**Figure S6: Yeast surface display screening of BSD1 variants against Flpp3 through expression and avidity-based enrichment.**

Binding profiles of the BSD1 library during expression, initial avidity, and enrichment avidity sorts. A distinct population of Flpp3-binding cells is observed starting from the initial avidity sort at 1 µM, with further enrichment seen in the enrichment avidity sort.


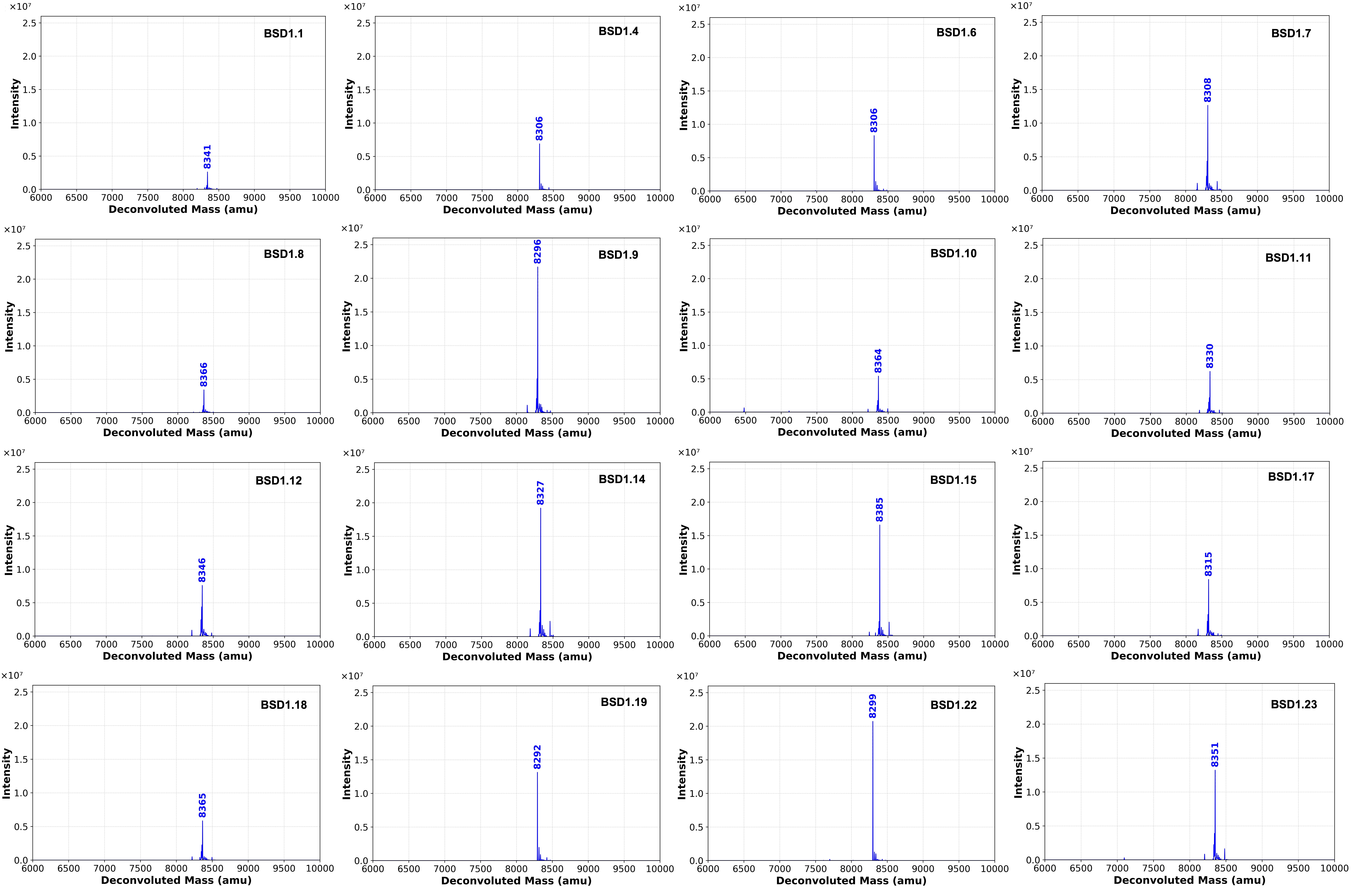


**Figure S7: Mass spectrometry analysis of expressed BSD1 variants.**

Deconvoluted electrospray ionization mass spectrometry (ESI-MS) spectra of selected BSD1 variants show single peaks corresponding to the expected molecular weights. All observed masses match the theoretical values, confirming successful expression and correct mass of the purified proteins.


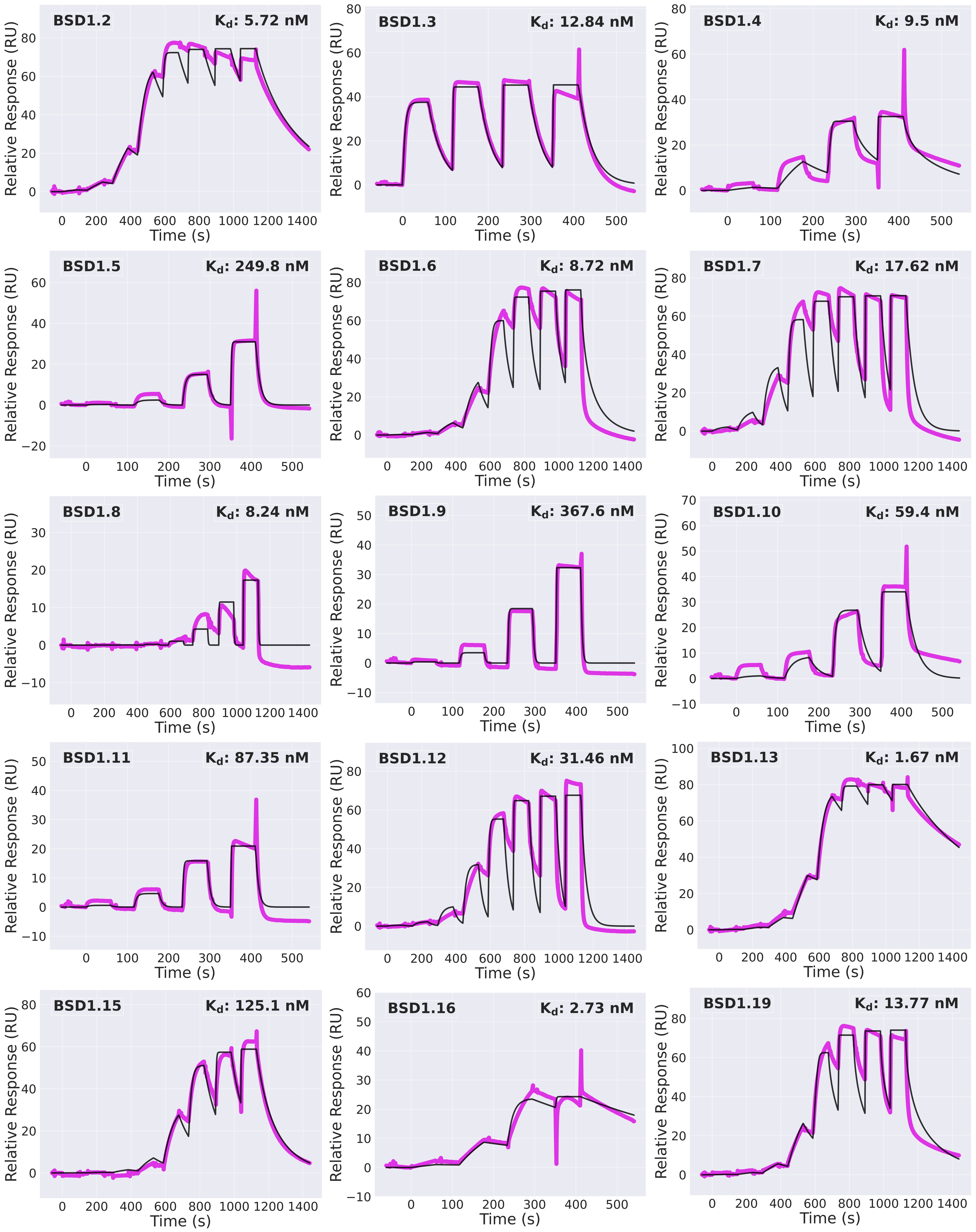


**Figure S8: SPR-based binding screen of additional BSD1 variants against Flpp3.**

SPR sensorgrams from a 4-point single cycle kinetics experiment (5-fold dilution, with highest concentrations ranging from 0.5 µM to 50 µM depending on the minibinder). Experimental data are shown in magenta, and global fits are shown with black lines.


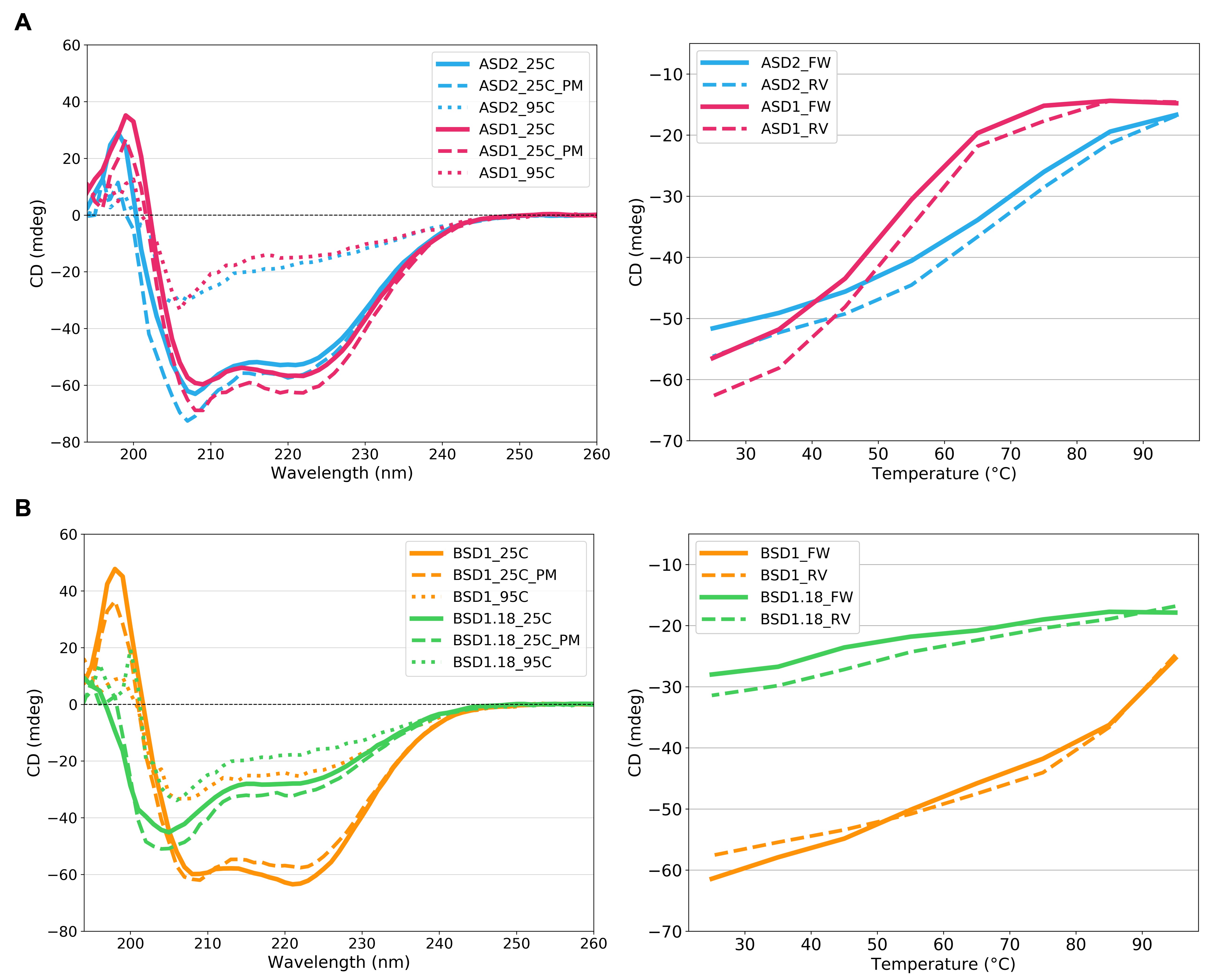


**Figure S9: Circular dichroism (CD) analysis of designed Flpp3 minibinders.**

(A) CD spectra between 195–260 nm for the top α-site binders and (B) β-site binders (panel B). (Left) Spectra recorded at 25°C, 95°C, and after cooling back to 25°C (labeled as 25C_PM). (Right) Thermal melt curves showing changes in the CD signal at 222 nm as the temperature was increased to 95°C (solid lines, labeled _FW) and then cooled back to 25°C (dashed lines, labeled _RV).


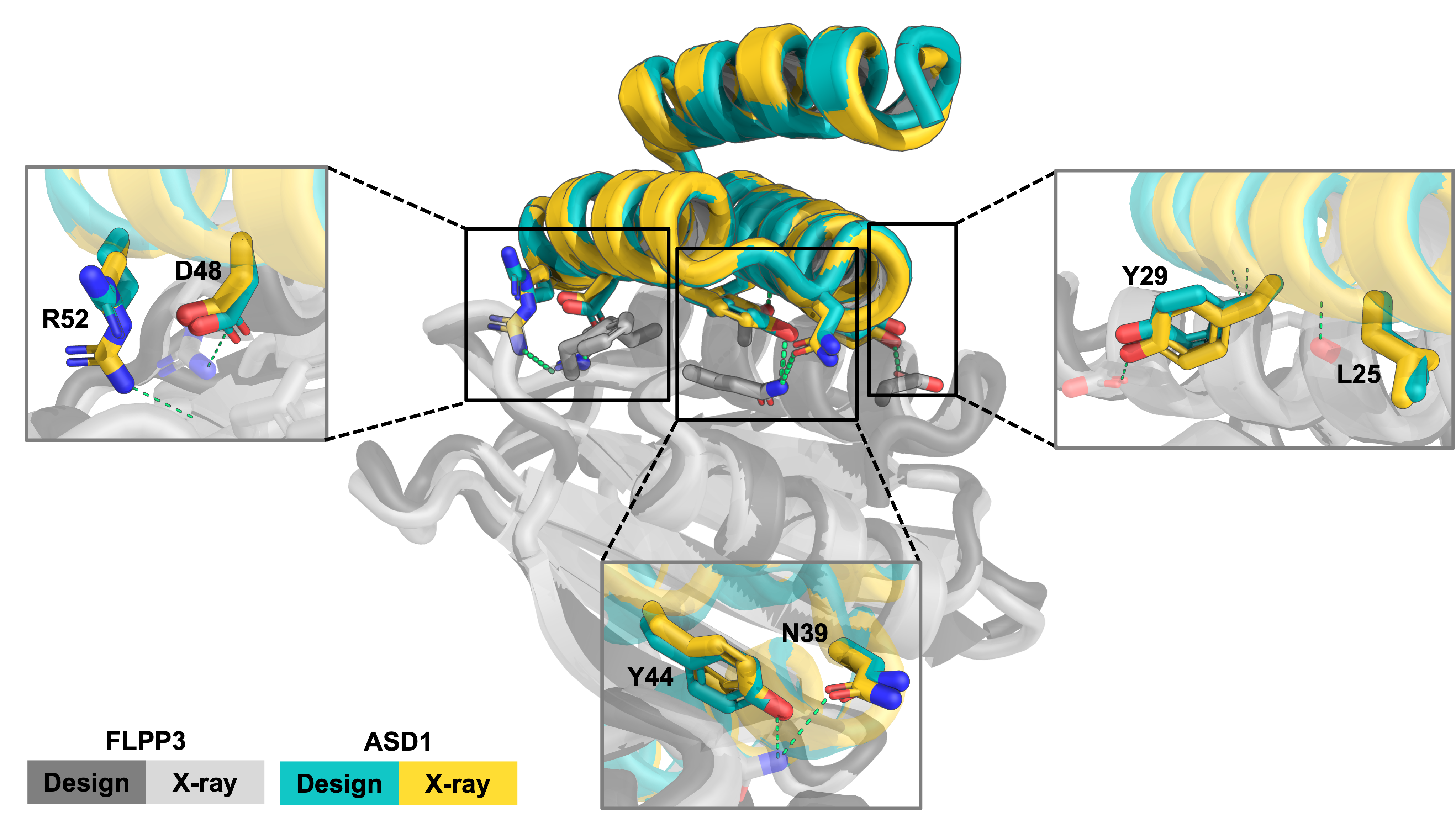


**Figure S10: X-ray crystal structure of Flpp3-ASD1 agrees closely with the design model**

Overall view of the ASD1 design model (teal) and X-ray structure (yellow) overlaid in complex with Flpp3 (gray). Insets show key interface residues (R52, D48, Y44, N39, Y29, L25), illustrating the close similarity in sidechain rotamers between the design model and the X-ray crystal structure.


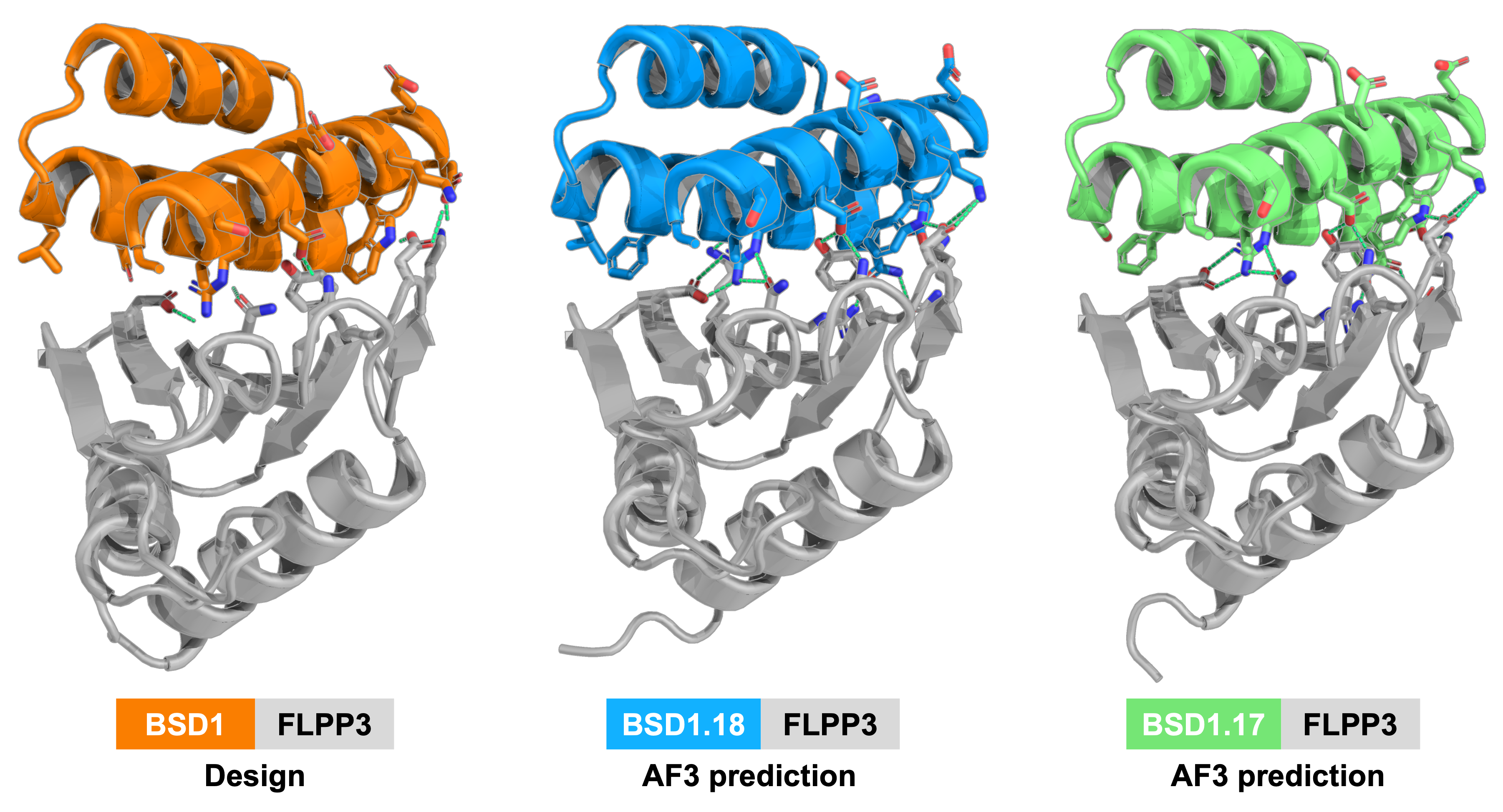


**Figure S11: Structural comparison of the original BSD1 design and AlphaFold3-predicted models of top binding variants.**

The de novo designed BSD1 miniprotein (left, orange) is shown in complex with Flpp3 (gray), alongside AlphaFold3-predicted structures of the two highest-affinity variants, BSD1.18 (middle, blue) and BSD1.17 (right, green). Both variants closely match the overall fold and binding mode of the original design, suggesting that the interface remains largely unchanged.

**
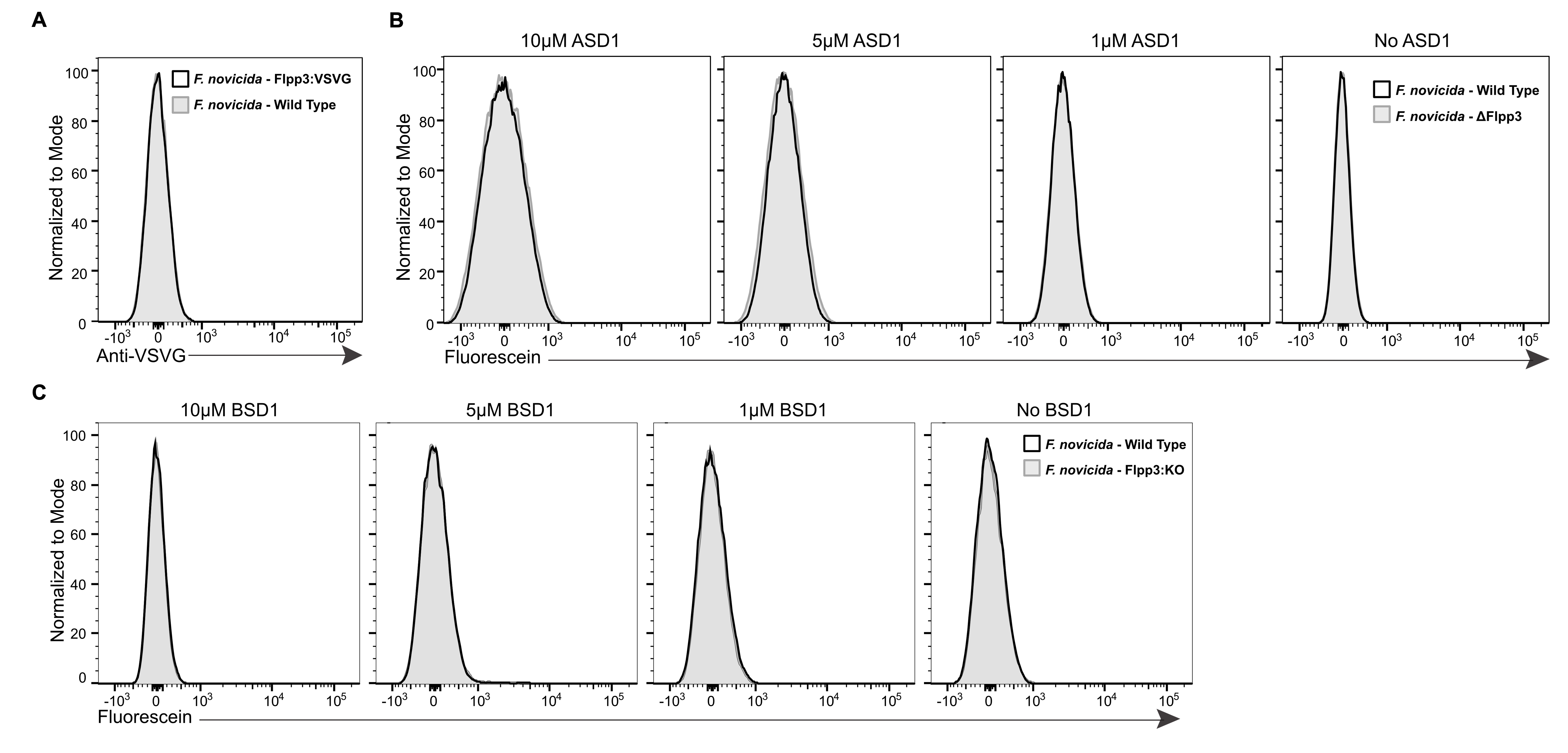
**

**Figure S12: Flow cytometry analysis of surface expression and minibinder binding in *F. novicida.***

(A) *F. novicida* strains expressing Flpp3-VSV-G were stained with an anti-VSV-G antibody followed by a FITC-labeled secondary antibody to assess surface expression. Wild-type (WT) bacteria were included as a control. (B) WT and Δ*flpp3* *F. novicida* strains were stained with FITC-labeled ASD1 minibinders at concentrations of 10 µM, 5 µM, 1 µM, and 0 µM. (C) WT and Flpp3 knockout (KO) strains were incubated with a biotinylated BSD1 minibinder and detected with streptavidin-phycoerythrin (SAPE). Minibinder concentrations were 10 µM, 5 µM, 1 µM, and 0 µM. Histograms represent fluorescence intensity normalized to mode.


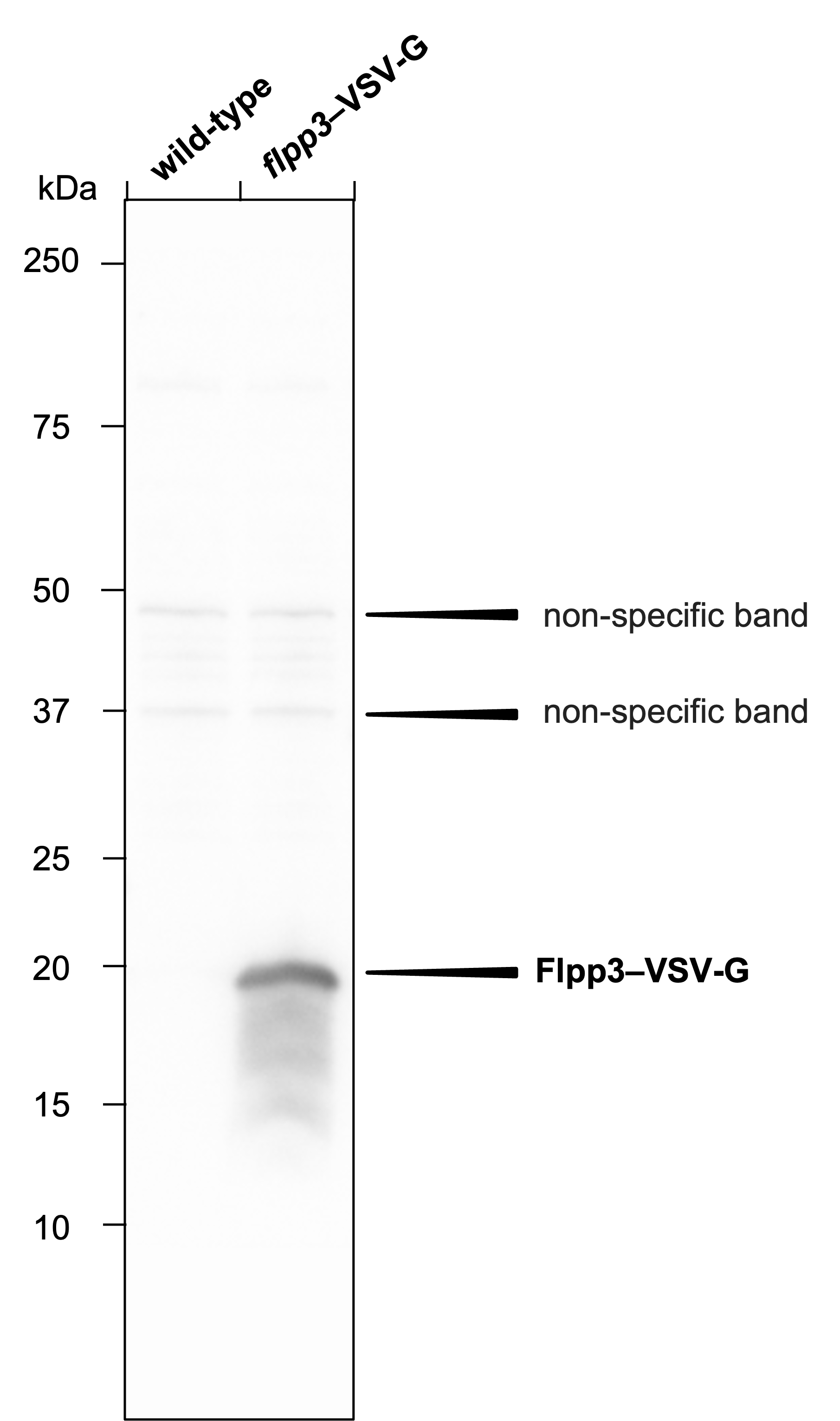


**Figure S13: Western blot analysis of *flpp3*–VSV-G expression in *F. novicida*.**

Whole-cell lysates from *F. novicida* wild-type and *flpp3*–VSV-G strains were probed with an anti–VSV-G antibody to detect expression of the Flpp3–VSV-G fusion protein. A strong band is observed in the *flpp3*–VSV-G lane, indicating successful expression. Molecular weights were estimated based on the Precision Plus Protein Standards Dual Color ladder (Bio-Rad Cat#1610374). Image shown is the chemiluminescent signal acquired using the iBright™ imaging system, with no contrast adjustments applied. Lane identities are labeled above the blot.

**Table S1: Oligonucleotides used in this study.**

| **Oligonucleotides or linear DNA fragments** | **Source** |
| --- | --- |
| F1_∆FTN_1382_BamHI (5' – 3') ATTCGAGCTCGGTACCCGGGGATCCTTGCTACGATGTTGTAATTGTTTATC | Integrated DNA Technologies |
| R1_∆FTN_1382 (5' – 3') GAATATTTCCTTTCATATTATTTATTGATATACATAGTTCAG | Integrated DNA Technologies |
| F2_∆FTN_1382 (5' – 3') TAATATGAAAGGAAATATTCGCTACGCTAATAC | Integrated DNA Technologies |
| R2_∆FTN_1382_PstI (5' – 3') GCCAAGCTTGCATGCCTGCAGTGTTTCTGGTACTAGCAATTTTG | Integrated DNA Technologies |
| Check1_∆FTN_1382 (5' – 3') CTGATAGAGTGCCTTGAGAATCC | Integrated DNA Technologies |
| Check2_∆FTN_1382 (5' – 3') ATGTCAGATCTGCATATACTGTAGC | Integrated DNA Technologies |
| F1_FTN_1382_VSVG_BamHI (5' – 3') ATTCGAGCTCGGTACCCGGGGATCCTCGTATGTATAAACCTGTTTCTC | Integrated DNA Technologies |
| R1_FTN_1382_VSVG (5' – 3') TTTTCCTAATCTATTCATTTCAATATCTGTATATGTATTAGCGTAGCGAATATTTC | Integrated DNA Technologies |
| F2_FTN_1382_VSVG (5' – 3') TATTGAAATGAATAGATTAGGAAAATAATATGTAAAACTTAAAACTTTTATATATGATTG | Integrated DNA Technologies |
| R2_FTN_1382_VSVG_PstI (5' – 3') GCCAAGCTTGCATGCCTGCAGAACAATCTTCGAAGCAAAATATG | Integrated DNA Technologies |
| Check1_FTN_1382_VSVG (5' – 3') GCCAGCATGCGACATTGATAAG | Integrated DNA Technologies |
| Check2_FTN_1382_VSVG (5' – 3') TTTAAGTTTTACATATTATTTTCCTAATCTATTCATTTC | Integrated DNA Technologies |

*****FTN_1382 corresponds to *flpp3*.

**Table S2:** **Data collection and refinement statistics**

|  | ***ASD1 - Flpp3 complex (PDB ID: 9NLT)*** |
| --- | --- |
| **Data collection** |  |
| Space group | *P 2_1_* |
| Cell dimensions |  |
| *a*, *b*, *c* (Å) | 36.17, 42.89, 54.05 |
| α, β, γ (°) | 90, 96.28, 90 |
| Resolution (Å) | 35.95 - 2.37 (2.50 - 2.37) |
| *R*_merge_ | 0.327 (1.475) |
| *I* / σ*I* | 4.3 (1.9) |
| Completeness (%) | 95.0 (99.9) |
| Redundancy | 6.3 (6.5) |
| **Refinement** |  |
| Resolution (Å) | 35.95 - 2.37 (2.56 - 2.37) |
| No. reflections | 6458 (1233) |
| *R*_work_ / *R*_free_ | 0.2218 (0.3330) /0.2688 (0.3825) |
| No. atoms |  |
| Protein | 1266 |
| Ligand/ion | 2 |
| Water | 11 |
| *B*-factors |  |
| Protein | 51 |
| Ligand/ion | 29 |
| Water | 38 |
| R.m.s. deviations |  |
| Bond lengths (Å) | 0.002 |
| Bond angles (°) | 0.460 |

*Single Crystal used for each data collection.

*Values in parentheses are for the highest-resolution shell.

**Table S3: Amino acid sequences of the Flpp3 minibinders**

| ***Name*** | ***Sequence*** |
| --- | --- |
| ASD1 | MEEKEKEFNEKLEELKKAKTEEEKLELAYECGLLAGEINDPKYYRALDEVARAK |
| ASD2 | GREEVEKLCEEAVKEKDEKKREELIRRAAEIAAGYNDQESLKLVWDAIEKIES |
| ASD3 | SKEEEDVELVLKEIDKLVADGQPEIAKIVAEKVVEHLEELGNPDLAKRVRDKLEEI |
| ASD4 | EVMEKVKKLCEEAEEAKKAGNWEKVEELMRKAGLVAGEAGDLEACQLVDKKAKELEE |
| BSD1 | LISSVLQDRLETAKKWAEEGDKENAKFLLESAKQLAELVGDEETVKECEELLKKI |
| BSD1.1 | LISSVLQDRLETAKKWAEEGDKENAKFLLESAK**F**LAELVGDEETVKECEELLKKI |
| BSD1.2 | LISSVLQDRLETAKKWAEEGDKENAKFLLESAK**Y**LAELVGDEETVKECEELLKKI |
| BSD1.3 | LISSVLQDRLETAKKWAEEGDKENAKFLLESAK**V**LAELVGDEETVKECEELLKKI |
| BSD1.4 | LISSVLQDRL**L**TAKKWAEEGDKENAKFLLESAKQLAELVGDEETVKECEELLKKI |
| BSD1.5 | LISSVLQDRLETAKKWA**N**EGDKENAKFLLESAKQLAELVGDEETVKECEELLKKI |
| BSD1.6 | LISSVLQDRLETAKKWA**L**EGDKENAKFLLESAKQLAELVGDEETVKECEELLKKI |
| BSD1.7 | LISSVLQDRLETAKKWAEEGDKENAKFLL**D**SAKQLAELVGDEETVKECEELLKKI |
| BSD1.8 | LISSVLQDRLETAKKWAEEGDKENAKFLLES**D**KQLAELVGDEETVKECEELLKKI |
| BSD1.9 | LISSVLQDRLETAKKWAEEGDKENAKFLLESAKQLAE**S**VGDEETVKECEELLKKI |
| BSD1.10 | LISSVLQDRLETAKKWAEEGDKENAKFLLESAKQLAELV**V**DEETVKECEELLKKI |
| BSD1.11 | LISSVLQDRLETAKKWAEEGDKENAKFLLESAKQLAELVGD**H**ETVKECEELLKKI |
| BSD1.12 | LISSVLQDRLETAKKWAEEGDKENAKFLLESAKQLAELVGDEETVKECEELLKK**H** |
| BSD1.13 | LISSVLQDRL**L**TAKKWAEEGDKENAKFLLESAK**F**LAELVGDEETVKECEELLKKI |
| BSD1.14 | LISSVLQDRLETAKKWAEEGDKENAKFLL**D**SAK**F**LAELVGDEETVKECEELLKKI |
| BSD1.15 | LISSVLQDRLETAKKWAEEGDKENAKFLLES**D**K**F**LAELVGDEETVKECEELLKKI |
| BSD1.16 | LISSVLQDRLETAKKWAEEGDKENAKFLLESAK**F**LAELVGD**H**ETVKECEELLKKI |
| BSD1.17 | LISSVLQDRLETAKKWAEEGDKENAKFLLESAK**F**LAE**S**VGDEETVKECEELLKKI |
| BSD1.18 | LISSVLQDRLETAKKWAEEGDKENAKFLLESAK**F**LAELVGDEETVKECEELLKK**H** |
| BSD1.19 | LISSVLQDRL**L**TAKKWAEEGDKENAKFLL**D**SAKQLAELVGDEETVKECEELLKKI |
| BSD1.20 | LISSVLQDRL**L**TAKKWAEEGDKENAKFLL**D**SAK**F**LAELVGDEETVKECEELLKKI |
| BSD1.21 | LISSVLQDRL**L**TAKKWAEEGDKENAKFLLESAK**F**LAELVGD**H**ETVKECEELLKKI |
| BSD1.22 | LISSVLQDRL**L**TAKKWAEEGDKENAKFLLESAK**F**LAE**S**VGDEETVKECEELLKKI |
| BSD1.23 | LISSVLQDRLETAKKWAEEGDKENAKFLL**D**SAK**F**LAELVGDEETVKECEELLKK**H** |
| BSD1.24 | LISSVLQDRL**L**TAKKWAEEGDKENAKFLL**D**SAKQLAELVGD**H**ETVKECEELLKKI |

**REFERENCES**

[30] [E. T. Boder, K. D. Wittrup, *Nat. Biotechnol.* **1997**, *15*, 553–557.](http://paperpile.com/b/NMKabq/fUm6E)

[31] [*FlowJo^TM^ Software*, **n.d.**](http://paperpile.com/b/NMKabq/qGdxc)

[32] [B. Dang, M. Mravic, H. Hu, N. Schmidt, B. Mensa, W. F. DeGrado, *Nat. Methods* **2019**, *16*, 319–322.](http://paperpile.com/b/NMKabq/7Cgdr)

[33] [A. Eshraghi, J. Kim, A. C. Walls, H. E. Ledvina, C. N. Miller, K. M. Ramsey, J. C. Whitney, M. C. Radey, S. B. Peterson, B. R. Ruhland, B. Q. Tran, Y. A. Goo, D. R. Goodlett, S. L. Dove, J. Celli, D. Veesler, J. D. Mougous, *Cell Host Microbe* **2016**, *20*, 573–583.](http://paperpile.com/b/NMKabq/N19RP)

[34] [H. E. Ledvina, K. A. Kelly, A. Eshraghi, R. L. Plemel, S. B. Peterson, B. Lee, S. Steele, M. Adler, T. H. Kawula, A. J. Merz, S. J. Skerrett, J. Celli, J. D. Mougous, *Cell Host Microbe* **2018**, *24*, 285–295.e8.](http://paperpile.com/b/NMKabq/qf5Yd)

[35] [Y. Wang, H. E. Ledvina, C. A. Tower, S. Kambarev, E. Liu, J. C. Charity, L. S. M. Kreuk, Q. Tang, Q. Chen, L. A. Gallagher, M. C. Radey, G. F. Rerolle, Y. Li, K. M. Penewit, S. Turkarslan, S. J. Skerrett, S. J. Salipante, N. S. Baliga, J. J. Woodward, S. L. Dove, S. B. Peterson, J. Celli, J. D. Mougous, *Cell Host Microbe* **2023**, *31*, 1359–1370.e7.](http://paperpile.com/b/NMKabq/UrRI7)

[36] [R. E. Chamberlain, *Appl. Microbiol.* **1965**, *13*, 232–235.](http://paperpile.com/b/NMKabq/B2hQ3)

[37] [U. K. Laemmli, *Nature* **1970**, *227*, 680–685.](http://paperpile.com/b/NMKabq/orgYx)

[38] [W. Kabsch, *Acta Crystallogr. D Biol. Crystallogr.* **2010**, *66*, 125–132.](http://paperpile.com/b/NMKabq/KK3x4)

[39] [M. D. Winn, C. C. Ballard, K. D. Cowtan, E. J. Dodson, P. Emsley, P. R. Evans, R. M. Keegan, E. B. Krissinel, A. G. W. Leslie, A. McCoy, S. J. McNicholas, G. N. Murshudov, N. S. Pannu, E. A. Potterton, H. R. Powell, R. J. Read, A. Vagin, K. S. Wilson, *Acta Crystallogr. D Biol. Crystallogr.* **2011**, *67*, 235–242.](http://paperpile.com/b/NMKabq/YGfOR)

[40] [A. J. McCoy, R. W. Grosse-Kunstleve, P. D. Adams, M. D. Winn, L. C. Storoni, R. J. Read, *J. Appl. Crystallogr.* **2007**, *40*, 658–674.](http://paperpile.com/b/NMKabq/UPglJ)

[41] [P. D. Adams, P. V. Afonine, G. Bunkóczi, V. B. Chen, I. W. Davis, N. Echols, J. J. Headd, L.-W. Hung, G. J. Kapral, R. W. Grosse-Kunstleve, A. J. McCoy, N. W. Moriarty, R. Oeffner, R. J. Read, D. C. Richardson, J. S. Richardson, T. C. Terwilliger, P. H. Zwart, *Acta Crystallogr. D Biol. Crystallogr.* **2010**, *66*, 213–221.](http://paperpile.com/b/NMKabq/pEK8d)

[42] [P. Emsley, K. Cowtan, *Acta Crystallogr. D Biol. Crystallogr.* **2004**, *60*, 2126–2132.](http://paperpile.com/b/NMKabq/dJQDB)

[43] [C. J. Williams, J. J. Headd, N. W. Moriarty, M. G. Prisant, L. L. Videau, L. N. Deis, V. Verma, D. A. Keedy, B. J. Hintze, V. B. Chen, S. Jain, S. M. Lewis, W. B. Arendall 3rd, J. Snoeyink, P. D. Adams, S. C. Lovell, J. S. Richardson, D. C. Richardson, *Protein Sci.* **2018**, *27*, 293–315.](http://paperpile.com/b/NMKabq/3cymm)

[44] [J. D. Hunter, *Comput. Sci. Eng.* **2007**, *9*, 90–95.](http://paperpile.com/b/NMKabq/JLHOV)

[45] [M. Waskom, *J. Open Source Softw.* **2021**, *6*, 3021.](http://paperpile.com/b/NMKabq/UCfXR)

[46] [S. B. Needleman, C. D. Wunsch, *J. Mol. Biol.* **1970**, *48*, 443–453.](http://paperpile.com/b/NMKabq/ZrFYA)
